# Identification, Classification, and Expression Analysis of *GRAS* Gene Family in *Malus domestica*

**DOI:** 10.3389/fphys.2017.00253

**Published:** 2017-04-28

**Authors:** Sheng Fan, Dong Zhang, Cai Gao, Ming Zhao, Haiqin Wu, Youmei Li, Yawen Shen, Mingyu Han

**Affiliations:** College of Horticulture, Northwest A&F UniversityYangling, China

**Keywords:** apple, *GRAS*, synteny, expression analysis, flower induction

## Abstract

*GRAS* genes encode plant-specific transcription factors that play important roles in plant growth and development. However, little is known about the *GRAS* gene family in apple. In this study, 127 *GRAS* genes were identified in the apple (*Malus domestica* Borkh.) genome and named *MdGRAS1* to *MdGRAS127* according to their chromosomal locations. The chemical characteristics, gene structures and evolutionary relationships of the *MdGRAS* genes were investigated. The 127 *MdGRAS* genes could be grouped into eight subfamilies based on their structural features and phylogenetic relationships. Further analysis of gene structures, segmental and tandem duplication, gene phylogeny and tissue-specific expression with ArrayExpress database indicated their diversification in quantity, structure and function. We further examined the expression pattern of *MdGRAS* genes during apple flower induction with transcriptome sequencing. Eight higher *MdGRAS* (*MdGRAS6, 26, 28, 44, 53, 64, 107*, and *122*) genes were surfaced. Further quantitative reverse transcription PCR indicated that the candidate eight genes showed distinct expression patterns among different tissues (leaves, stems, flowers, buds, and fruits). The transcription levels of eight genes were also investigated with various flowering related treatments (GA_3_, 6-BA, and sucrose) and different flowering varieties (Yanfu No. 6 and Nagafu No. 2). They all were affected by flowering-related circumstance and showed different expression level. Changes in response to these hormone or sugar related treatments indicated their potential involvement during apple flower induction. Taken together, our results provide rich resources for studying *GRAS* genes and their potential clues in genetic improvement of apple flowering, which enriches biological theories of *GRAS* genes in apple and their involvement in flower induction of fruit trees.

## Introduction

Transcription factors function as trans-acting factors, combining with specific *cis*- elements in eukaryotic gene promoter regions to regulate plant growth and development.

*GRAS* genes encode a family of plant-specific transcription factors. The GRAS name derives from the first letters of the first identified three members, including *GAI* (gibberellic acid insensitive), *RGA* (repressor of *GA1-3* mutant), and *SCR* (scarecrow) (Bolle, 2004). In plants, proteins in the GRAS family consist of 400–700 amino acids with a conserved GRAS carboxyl terminus comprising several typical structures, including seven leucine-repeat domains (LHRI), a VHIID domain, seven leucine-repeat II domains (LHRII), and PFYRE and SAW motifs (Hirsch et al., 2009). Bacteria also harbor GRAS-like proteins, which may function as methylases (Zhang et al., 2012). In recent years, Many *GRAS* genes have also been identified in a variety of plant species, including *Prunus mume, Populus*, grape, tomato, Chinese cabbage, and castor bean (Tian et al., 2004; Song et al., 2014; Huang et al., 2015; Lu et al., 2015; Grimplet et al., 2016; Xu W. et al., 2016). The *GRAS* family, which has 33 members in *Arabidopsis thaliana* and 57 members in rice (*Oryza sativa*), can be divided into eight subfamilies: SCL3, SHR, PAT1, LISCL, DELLA, SCR, LS, and HAM (Hirsch et al., 2009).

Recent studies have revealed that the N-termini of GRAS proteins play important roles in their specific functions. The N-termini contain intrinsically disordered regions (IDRs); thus, GRAS proteins are also known as intrinsically disordered proteins (IDPs) (Sun et al., 2011). Several studies have focused on the structures and functions of the IDRs in the N-termini of GRAS proteins (Sun et al., 2010, 2011, 2012). Different subfamilies have different types and numbers of molecular recognition features in their N-termini (Sun et al., 2010, 2011, 2012). For instance, the DELLA subfamily has three molecular recognition features in its IDR at the N terminus: DELLA, VHYNP, and L(R/K) XI. Protein motifs at the C-terminus include the LHR I, HIID, LHRII, PFYRE, and SAW motifs (Pysh et al., 1999).

The functions of *GRAS* family genes have been studied in recent years; there is a large body of evidence that the *GRAS* genes family play important roles in various biological processes in many different plant species. They are involved in diverse processes related to root meristem development, axillary bud outgrowth, phytochrome signaling pathways, nodular signal transduction, and stress adaptation, especially in the gibberellins (GA)-signaling pathway (Ait-ali et al., 2003; Greb et al., 2003; Heckmann et al., 2006; Torres-Galea et al., 2006; Koizumi et al., 2012; Park et al., 2013). DELLA proteins are negative regulators of the GA signal, and have been shown to repress growth in many different plant species including *Arabidopsis* (AtGAI, AtRGA, AtRGL1, AtRGL2, and AtRGL3), *O. sativa* (OsSLR1), *Zea mays* (ZmD8), *Malus domestica* (MdRGL2a), and *Vitis vinifera* (VvGAI) (Foster et al., 2007; Vandenbussche et al., 2007; Aleman et al., 2008; Dai and Xue, 2010). Various studies have shown that the GA-GID1-DELLA complex and ubiquitin ligase E3 play important roles in GA signaling, and that *SCR* functions in the development of roots and above-ground organs in *Arabidopsis* (Zhang et al., 2011; Koizumi et al., 2012). *PhHAM* (*Petunia hybrid*), a petunia hairy meristem gene, was shown to be necessary to maintain the apical meristem in petunia, and the petunia recessive mutant *phham* (*ham*-*B4281*) produced fewer flowers than wild type (Stuurman et al., 2002). *SCL13* was shown to positively regulate phyB, and the *scl13* mutant showed impaired sensitivity in *Arabidopsis* (Torres-Galea et al., 2006). Several studies have shown that some *GRAS* genes are up-regulated under stress conditions in *Arabidopsis*, rice and tobacco (Day et al., 2003; Torres-Galea et al., 2006; Czikkel and Maxwell, 2007). Among all the biological processes, their flowering related characterizations and functions have been researched in some model plants. On the one hand, several *GRAS* genes are associated with the flowering phenotype. For example, in tomato, *Ls* (*Later Suppressor*), defined as another *GRAS* gene, its mutant *ls* showed lower numbers of inflorescence (Schumacher et al., 1999; Bolle, 2004). In *Arabidopsis*, the LS subfamily gene *AtLAS* and the SHR subfamily gene *AtSHR* were involved in shoot apical meristem. The Ls subfamily gene *AtSCL26* and the HAM subfamily genes *AtSCL6* and *AtSCL27* were highly expressed in flowers (Bolle, 2004); the *rga* and gai mutant also showed earlier flowering in Arabidopsis (Galvao et al., 2012). On the other hand, *GRAS* genes can integrate or regulate flowering related genes such as *SPL* (*SQUAMOSA PROMOTER BINDING PROTEIN-LIKE*), *LFY* (*LEAFY*), and *AP1* (*APETALA1*) as well as *FT* (*FLOWERING LOCUS T*). They all acted positive roles and intergrated various signals in flower induction, of which *FT* and *SPL* affected flowering time; while *LFY* and *AP1* belonged to flower meristem identify genes. Several *GRAS* genes can be recruited and integrated with SPL proteins to regulate *LFY* and *AP1* expression in *Arabidopsis* (Yamaguchi et al., 2014). Additionally, the PAT and SCL subfamilies of *GRAS* genes were both reported involved in photosignal in many plants, it influenced the expression of *phyA* (phytochrome A), a phytochrome A signal transduction gene, which caused to the change of the circadian clock and expression of *FT* and other flowering genes (Bolle et al., 2000; Torres-Galea et al., 2006; Li et al., 2014). However, little is known about the *GRAS* family of transcription factors in woody fruit trees, such as its expression in response to flower induction in apple.

Apple is one of the most widely cultivated and economically important fruit trees in the temperate regions of the world. In apple production, flower induction and flower buds formation sustain two growing seasons. Flower induction is a key stage, and always limits fruit yield (Guitton et al., 2012). Apple (*Malus domestica* Borkh.) cv. “Fuji” is one of the most popular cultivars, and it accounts for 65% planting areas in China. “Fuji” has trouble in flowering induction. Thus, characteristic the molecular mechanism of apple flower induction is necessary. Flower induction was affected by environmental and internal factors (Guitton et al., 2012; Xing et al., 2014, 2015; Fan et al., 2016). Sugar and hormones were involved in flower induction (Guitton et al., 2012; Xing et al., 2014, 2015; Fan et al., 2016; Li et al., 2016; Zhang et al., 2016). Among all kinds of hormones, including auxin, cytokinins, abscisic acid, GA and other hormones. GA was defined the most important hormones in regulating flower induction and GA pathway was recognized as one of the flowering pathways (Guitton et al., 2012; Porri et al., 2012; Li et al., 2016; Zhang et al., 2016). 6-BA and sucrose, which can promote flower bud differentiation, were also important and widely used (Xing et al., 2015; Li et al., 2016). Several flowering-related genes families have been identified and characterized in the apple genome, including *SPL, IDD*, and *MADX-box* (Li et al., 2013; Kumar et al., 2016; Fan et al., 2017). *GRAS* genes were reported to partially or entirely involve in flower induction in various plants (Hynes et al., 2003; Foster et al., 2007; Vandenbussche et al., 2007; Galvao et al., 2012; Yamaguchi et al., 2014). Little is known about *MdGRAS* genes and their potential involvement in apple flower induction. The recently published genome sequence of apple (Velasco et al., 2010) has made it possible to characterize the structures, functions, evolution of apple *GRAS* genes and their response to flower induction. Moreover, it is also available to utilize candidate *MdGRAS* genes for further genetic improvement aiming at flowering induction.

In this study, we performed a genome-wide survey to identify *GRAS* genes in the apple genome. A systematic analysis including gene classification, gene characterization, gene structures and gene phylogenies were determined. We further investigated their expression levels of all the 127 putative *MdGRAS* genes among different tissues and varieties during apple growth and development with ArrayExpress database. Moreover, eight *MdGRAS* genes were further analyzed.

We investigated their expression levels of the candidate eight genes among different tissues (stems, leaves, flowers, fruits, and buds), different flowering-related treatments (GA_3_, 6-BA and sugar_)_ and different flowering varieties (Yanfu No. 6 and Nagafu No. 2). Changes in response to these flowering-related treatments indicated their potential involvement in apple flower induction. Our results provided basic clues for further analyses of *MdGRAS* in apple growth and development.

## Materials and methods

### Identification of *GRAS* genes in apple

Hidden Markov Model (HMM) searches (Finn et al., 2011) were performed to identify *GRAS* genes in the apple genome. The HMM profiles (PF03514) were downloaded from the Pfam database (http://pfam.sanger.ac.uk). Additionally, AtGRAS protein sequences were used as queries for BLASTP search against the Apple Genome Database (https://www.rosaceae.org/) with an *e*-value < 1e-4 to identify further possible members of *GRAS* gene as previous investigation (Li et al., 2011; Jia et al., 2013; Cui et al., 2015; Xu J. N. et al., 2016). *Arabidopsis* GRAS genes were downloaded from TAIR (http://www.arabidopsis.org/). Finally, genes with the same accession number were removed. All the identified GRAS genes were further manually checked for the GRAS domain.

### Gene structures and locations determination

Annotations for candidate *GRAS* genes were obtained from the Genome Database for Rosaceae (GDR) (http://www.rosaceae.org), and chromosome locations were retrieved from these annotations. MapDraw was used to show the accurate locations of the genes on each chromosome. The online software Gene Structure Display Server (GSDS: http://gsds.cbi.pku.edu.cn) was used to generate the exon-intron structures of all the *GRAS* genes.

### Chemical characteristics, elements in the promoters and phylogenetic analysis

Prosite ExPaSy (http://web.expasy.org/protparam/) was used to predict the potential chemical characteristics of the *MdGRAS* genes. A phylogenetic tree was generated using MEGA 6.06 with the Maximum Likelihood (ML) method and 1000 bootstrap replications. The upstream of 1.5 Kb were used for *cis*-elements in the promoters of the candidate *MdGRAS* genes. PlantCARE software (http://bioinformatics.psb.ugent.be/webtools/plantcare/html/) was used for searching regulatory elements.

### Tandem duplication and synteny analysis

Tandem duplication and synteny analysis were conducted using Circos v. 0.63 (http://circos.ca/). Tandem duplication of the *MdGRAS* genes was identified according to their physical locations on individual chromosomes in the apple genome. Adjacent homologous *GRAS* genes on the same apple chromosome with no more than one intervening gene were considered tandem duplicates. The Plant Genome Duplication Database (http://chibba.agtec.uga.edu/duplication/) was used to identify syntenic blocks.

### Plant material

In this study, 72 uniform 6-year-old “Fuji”/T337/*Malus robusta* Rehd. apple trees were randomly divided into three groups: 18 were treated with GA_3_, 18 were treated with sucrose, 18 were treated with 6-BA and 18 were sprayed with water as a control. These tress were grown at the experimental orchard of Northwest A& F University in Yangling (108°04′ E, 34°16′ N), China. And the orchard was well managed. Each group consisted of three blocks, with three replicates. The experiment was conducted from 30 to 70 days after full bloom (DAFB) in 2015. (1) GA_3_ treatment was performed as described by Zhang et al. (2016) with a slight modification: 700 mg L^−1^ GA_3_ (Sigma, Deisenhofen, Germany) was sprayed once on a clear morning at 30 DAFB (May 9). (2) 300 mg L^−1^ 6-BA (Sigma, Deisenhofen, Germany) was also sprayed on a clear morning at 30 DAFB (May 9). (3)For sugar treatment, trees were sprayed twice on clear mornings at 30 and 37 DAFB (May 9 and May 16) with 15,000 mg L^−1^ and 20,000 mg L^−1^ sucrose. All treatments were performed on the whole plant and were applied with a low-pressure hand-wand sprayer. Terminal buds on current-year spurs (<5 cm), which were chosen according to our previous studies (Xing et al., 2014, 2015; Li et al., 2016; Zhang et al., 2016), were collected into liquid nitrogen at 30, 40, 50, 60, and 70 DAFB, and then stored at −80°C for use in gene expression analyses.

Additionally, buds from two apple varieties (“Yanfu No. 6” and “Nagafu No. 2”) with contrasting flowering rates were collected at 30, 40, 50, 60, and 70 DAFB from 18 uniform 6-year-old trees in 2015. “Yanfu No. 6” is a spontaneous mutant of “Nagafu No. 2.” It develops more flower buds than “Nagafu No. 2.” “Yanfu No. 6” always exhibits a higher proportion of spurs, shorter internodes, bigger buds and more flowers. Terminal buds on current-year spurs (<5 cm) were collected as described above.

Different organs were also collected for tissue-specific expression analysis. Flowers were collected at full blossom on April 9 in 2015. Stems were collected from new shoots with a diameter of 2–3 mm. Mature leaves were collected from the adjacent buds. Fruits were collected with a diameter of 2–3 cm. All tissues were immediately frozen in liquid nitrogen and stored at −80°C for gene expression analysis. Additionally, the online ArrayExpress database (https://www.ebi.ac.uk/arrayexpress/, E-GEOD-42873) was also employed to investigate their expression patterns among different apple varieties and tissues.

### Flowering rate analysis

Flowering rate was also investigated among different exogenous treatment and two different flowering varieties. One hundred twenty terminal buds on short shoots which were less than 5 cm and no fruits on them were tagged randomly at 15 DAFB. The number of flowers was counted of the tagged shorts as finally flowering rate. And it was investigated at full blossom on April 10 in 2016.

### RNA extraction and cDNA synthesis

Total RNA was extracted from plant tissue samples using the cetyltrimethyl ammonium bromide (CTAB) method with slight modifications (Gambino et al., 2008). Briefly, 900 μL extraction buffer (2% CTAB, 2.5% PVP-40, 2 M NaCl, 100 mM Tris-HCl [pH 8.0], 25 mM EDTA [pH 8.0], and 2% β-mercaptoethanol) was preheated at 65°C and added to 2-mL microcentrifuge tubes just before use. Samples containing 200 mg of bud tissue stored at −80°C were ground to a powder, added to the tubes, and mixed with extraction buffer. After shaking and inverting each tube vigorously for 5 min and incubating at 65°C for 30 min, an equal volume of chloroform:isoamyl alcohol (24:1, v/v) was added. Each tube was shaken and inverted vigorously and then centrifuged at 12,000 × *g* for 10 min at 4°C. The supernatant was collected into a new tube and re-extracted with an equal volume of chloroform:isoamyl alcohol (24:1, v/v). The resulting supernatant was then transferred into a new 2-mL tube and LiCl (3 M final concentration) was added. The mixture was incubated at −20°C for 4 h and the RNA was selectively pelleted by LiCl after centrifugation at 18,000 × *g* for 20 min at 4°C. The pellet was resuspended in 500 μL of SSTE buffer (10 mM Tris-HCl [pH 8.0], 1 mM EDTA [pH 8.0], 1% SDS, and 1 M NaCl) preheated to 65°C and an equal volume of chloroform:isoamyl alcohol. The mixture was then centrifuged at 12,000 × *g* for 10 min at 4°C. The supernatant was transferred to a new microcentrifuge tube, and the RNA was precipitated with 2.5 volumes of cold ethanol at −80°C for at least 30 min and centrifuged at 1,200 × *g* for 20 min at 4°C. Finally, the pellets were washed with 70% ethanol and resuspended in diethylpyrocarbonate-treated water.

Total RNA integrity was verified by running the samples on 2% agarose gels. First-strand cDNA was synthesized from 1 μg of total RNA using a PrimeScript RT Reagent kit with gDNA Eraser (Takara Bio, Shiga, Japan) following the manufacturer's instructions.

### Expression analysis by qRT-PCR

The expression levels of all identified *MdGRAS* genes were analyzed by qRT-PCR using primer pairs designed with Primer 5.0 (Table S1). The real-time PCR assay mix (20 μL) consisted of 2-μL cDNA samples (diluted 1:8), 10 μL 2 × SYBR Premix ExTaq II (Takara Bio), 0.8 μL of each primer (10 μM) (Table 1), and 6.4 μL distilled deionized H_2_O. Each PCR assay was run on an iCycler iQ Real Time PCR Detection System (Bio-Rad) with an initial denaturation at 95°C for 3 min, followed by 40 cycles at 94°C for 15 s, 62°C for 20 s, and 72°C for 20 s. The resulting fragments were subjected to melting-curve analysis to verify the presence of gene-specific PCR products. The melting-curve analysis was performed directly after real-time PCR and included an initial step of 94°C for 15 s, followed by a constant increase from 60° to 95°C at a 2% ramp rate. The apple *EF-1*α gene (GenBank accession no. DQ341381) was used as an internal control to normalize all mRNA expression levels. The 2^−ΔΔCt^ method was used to calculate the relative amount of template present in each PCR amplification mixture (Livak and Schmittgen, 2001).

**Table 1:** **List of ***MdGRAS*** genes and their chemical characteristics**.

**Gene name**	**Gene locus^a^**	**Location**	**CDS (bp)**	**Peptide (aa)**	**Strand**	**MW^b^ (kDa)**	**GRAVY^c^**	**PI^d^**	**II^e^**	**AI^f^**
*MdGRAS1*	MDP0000135256	chr1:16537486.16539435	930	309	−	33.36	−0.011	9.33	42.29	96.60
*MdGRAS2*	MDP0000252300	chr1:16538919.16544966	2,550	849	−	93.62	−0.547	8.10	52.08	75.54
*MdGRAS3*	MDP0000119183	chr1:18011215.18015301	1,356	451	−	50.28	−0.263	6.33	43.93	82.20
*MdGRAS4*	MDP0000825698	chr2:3727360.3729003	1,644	547	−	61.13	−0.316	10.01	59.37	81.72
*MdGRAS5*	MDP0000134341	chr2:3744608.3746247	1,572	523	−	57.30	−0.156	5.70	45.66	85.64
*MdGRAS6*	MDP0000256486	chr2:12924073.12925887	1,815	604	−	66.48	−0.378	5.13	52.06	78.86
*MdGRAS7*	MDP0000139971	chr2:13285923.13292998	2,331	776	−	86.60	−0.126	6.08	45.09	90.70
*MdGRAS8*	MDP0000247514	chr2:17606743.17608173	1,431	476	−	53.23	−0.069	5.67	43.06	90.99
*MdGRAS9*	MDP0000165587	chr2:18027038.18028561	1,524	507	−	56.88	−0.392	5.22	48.32	69.29
*MdGRAS10*	MDP0000859087	chr2:25937193.25938899	1,707	568	−	63.23	−0.446	5.02	44.23	74.00
*MdGRAS11*	MDP0000159341	chr3:4321397.4322773	1,377	458	+	51.96	−0.272	6.07	54.41	92.64
*MdGRAS12*	MDP0000194661	chr3:7771083.7773134	2,052	683	−	77.70	−0.645	5.52	53.63	74.64
*MdGRAS13*	MDP0000796348	chr3:7777819.7780444	2,160	719	−	82.05	−0.547	6.90	42.05	75.80
*MdGRAS14*	MDP0000194663	chr3:7781197.7788706	5,787	1928	−	218.53	−0.568	5.83	49.45	72.05
*MdGRAS15*	MDP0000247940	chr3:7827530.7831637	2,715	904	+	101.59	−0.540	5.38	50.48	71.38
*MdGRAS16*	MDP0000306154	chr3:20678219.20679634	1,365	454	−	50.87	−0.125	6.16	40.19	92.64
*MdGRAS17*	MDP0000793615	chr3:25279993.25282044	2,052	683	+	75.01	−0.418	5.64	52.31	77.42
*MdGRAS18*	MDP0000205622	chr3:25473680.25476670	2,007	668	−	74.17	−0.399	5.61	48.56	80.90
*MdGRAS19*	MDP0000835454	chr3:32975889.32976838	876	291	−	32.90	−0.713	5.40	41.66	68.38
*MdGRAS20*	MDP0000300311	chr3:33027149.33029331	735	244	−	27.06	0.038	8.86	43.86	90.66
*MdGRAS21*	MDP0000259453	chr3:33044014.33045333	1,188	395	−	43.95	−0.304	5.87	40.29	84.43
*MdGRAS22*	MDP0000420150	chr3:33053754.33054086	333	110	+	12.55	0.305	4.53	25.59	119.73
*MdGRAS23*	MDP0000141505	chr3:33061403.33061960	558	185	+	21.06	0.094	6.82	48.488	92.70
*MdGRAS24*	MDP0000465513	chr3:33061932.33063815	1,884	627	−	70.91	−0.263	5.50	41.06	81.31
*MdGRAS25*	MDP0000232659	chr3:33105946.33112977	3,024	1007	−	113.57	−0.535	7.10	50.65	73.76
*MdGRAS26*	MDP0000147173	chr4:915116.916492	1,377	458	−	51.30	−0.285	5.12	45.39	83.01
*MdGRAS27*	MDP0000396037	chr4:3510983.3512255	993	330	+	36.34	−0.308	5.33	48.90	67.45
*MdGRAS28*	MDP0000945004	chr4:4693449.4695500	2,052	683	−	74.98	−0.407	5.69	52.03	77.85
*MdGRAS29*	MDP0000241048	chr4:12671528.12673775	1,668	555	−	61.88	−0.387	5.98	49.78	79.89
*MdGRAS30*	MDP0000228483	chr4:16466303.16467886	1,584	527	−	59.59	−0.320	4.82	50.99	76.94
*MdGRAS31*	MDP0000317825	chr4:16469757.16471367	1,611	536	−	60.75	−0.309	4.67	53.32	75.67
*MdGRAS32*	MDP0000419045	chr4:18100227.18103427	2,490	829	−	88.67	−0.308	5.82	59.22	73.84
*MdGRAS33*	MDP0000254936	chr5:411761.422370	3,600	1199	+	133.82	−0.316	6.64	39.77	79.76
*MdGRAS34*	MDP0000151144	chr5:3168395.3171022	2,400	799	−	87.03	−0.267	6.03	57.31	76.92
*MdGRAS35*	MDP0000242829	chr5:10558101.10559735	1,635	544	+	60.88	−0.244	4.83	46.59	83.75
*MdGRAS36*	MDP0000230994	chr5:15148360.15151129	1,572	523	−	58.94	−0.320	6.24	48.46	82.26
*MdGRAS37*	MDP0000566079	chr5:22506172.22506504	330	110	−	12.50	−0.232	6.04	44.89	80.64
*MdGRAS38*	MDP0000243351	chr6:9882603.9884659	1,824	607	+	66.98	−0.509	5.19	53.22	72.49
*MdGRAS39*	MDP0000915100	chr6:9882928.9884982	2,055	694	+	75.47	−0.403	5.56	51.47	78.03
*MdGRAS40*	MDP0000322003	chr6:9923402.9927244	981	326	+	36.85	−0.214	4.91	43.91	88.50
*MdGRAS41*	MDP0000124576	chr6:16659904.16660845	567	188	−	21.11	−0.162	6.36	33.54	82.39
*MdGRAS42*	MDP0000128964	chr6:16680332.16681280	804	267	+	29.94	−0.114	7.19	29.99	84.72
*MdGRAS43*	MDP0000206202	chr6:23034606.23035559	954	317	−	35.85	−0.707	5.68	39.04	67.41
*MdGRAS44*	MDP0000159531	chr7:22551974.22553587	1,614	537	−	59.25	−0.215	5.34	55.11	85.72
*MdGRAS45*	MDP0000315706	chr7:24383528.24394942	2,817	938	−	103.28	−0.432	6.41	47.36	75.69
*MdGRAS46*	MDP0000275704	chr8:10371640.10374024	2,385	794	−	86.86	−0.327	6.05	55.01	76.76
*MdGRAS47*	MDP0000287321	chr8:18091272.18096730	1,827	608	+	67.24	−0.270	9.15	46.58	87.42
*MdGRAS48*	MDP0000441376	chr9:562753.565977	2,091	696	−	77.38	−0.406	5.73	64.97	73.22
*MdGRAS49*	MDP0000863616	chr9:6708558.6709889	1,332	443	−	49.06	−0.131	5.19	49.04	93.05
*MdGRAS50*	MDP0000804652	chr9:8891282.8892943	1,662	553	−	62.32	−0.346	5.74	49.48	78.66
*MdGRAS51*	MDP0000921788	chr9:9424971.9428298	2,073	690	−	77.57	−0.312	8.85	47.18	80.71
*MdGRAS52*	MDP0000203826	chr9:22347041.22348471	1,431	476	+	54.20	−0.347	6.31	41.21	89.33
*MdGRAS53*	MDP0000181482	chr9:29373931.29376390	1,938	645	+	70.41	−0.258	4.76	39.14	81.19
*MdGRAS54*	MDP0000727836	chr10:1113520.1115259	1,740	579	−	64.46	−0.448	5.03	40.79	75.80
*MdGRAS55*	MDP0000169115	chr10:5639948.5641363	1,416	471	+	53.93	−0.406	6.31	44.39	89.87
*MdGRAS56*	MDP0000512470	chr10:5640084.5641499	1,416	471	+	53.93	−0.406	6.31	44.39	89.87
*MdGRAS57*	MDP0000307619	chr10:18265808.18267481	1,464	487	−	54.88	−0.243	6.07	46.99	86.12
*MdGRAS58*	MDP0000288971	chr10:22109238.22112956	831	276	−	31.19	−0.097	5.27	47.65	91.45
*MdGRAS59*	MDP0000162468	chr10:26355418.26356833	1,416	471	+	53.93	−0.406	6.31	44.39	89.87
*MdGRAS60*	MDP0000443053	chr10:29148732.29150108	1,377	458	−	51.67	−0.211	5.81	52.99	90.74
*MdGRAS61*	MDP0000607785	chr10:32638991.32646762	1,821	606	+	67.87	−0.369	6.58	43.64	76.73
*MdGRAS62*	MDP0000715138	chr11:2058625.2059440	717	238	−	26.65	−0.444	5.12	46.87	68.49
*MdGRAS63*	MDP0000389025	chr11:4337648.4339036	1,389	462	+	52.60	−0.243	6.03	58.05	94.35
*MdGRAS64*	MDP0000264347	chr11:8307859.8309883	2,025	674	+	76.62	−0.616	5.40	48.74	74.32
*MdGRAS65*	MDP0000287039	chr11:8329524.8331601	1,233	410	+	47.21	−0.377	7.20	38.27	78.71
*MdGRAS66*	MDP0000851230	chr11:8330639.8332645	2,007	668	−	76.04	−0.609	5.96	45.35	73.13
*MdGRAS67*	MDP0000289783	chr11:8333605.8335590	1,986	661	−	75.26	−0.570	6.47	54.65	70.67
*MdGRAS68*	MDP0000287040	chr11:8334218.8339685	4,134	1377	+	155.67	−0.517	6.02	52.42	72.45
*MdGRAS69*	MDP0000289784	chr11:8336934.8339074	2,100	699	−	78.44	−0.478	5.90	47.91	73.96
*MdGRAS70*	MDP0000287041	chr11:8340645.8342651	2,007	668	+	76.04	−0.609	5.96	45.35	73.13
*MdGRAS71*	MDP0000250943	chr11:8344394.8346472	2,076	692	−	78.26	−0.562	5.89	46.69	71.03
*MdGRAS72*	MDP0000827722	chr11:8398699.8400897	2,199	732	+	82.40	−0.519	5.80	44.56	72.45
*MdGRAS73*	MDP0000453000	chr11:8405492.8408822	3,054	1017	+	114.99	−0.545	5.86	46.75	72.50
*MdGRAS74*	MDP0000227056	chr11:8417502.8420917	2,607	868	+	97.64	−0.580	6.17	52.53	65.66
*MdGRAS75*	MDP0000236041	chr11:8419367.8421957	2,313	770	+	88.11	−0.491	8.69	45.15	77.70
*MdGRAS76*	MDP0000581417	chr11:8434126.8436324	2,199	732	−	82.40	−0.519	5.80	44.56	72.45
*MdGRAS77*	MDP0000822651	chr11:10148161.10149699	1,539	512	−	57.41	−0.381	5.33	47.06	68.22
*MdGRAS78*	MDP0000301368	chr11:20713600.20715024	1,425	474	+	52.98	−0.156	5.95	40.62	94.49
*MdGRAS79*	MDP0000809337	chr11:25863922.25865673	1,752	583	+	64.77	−0.378	5.94	51.14	84.32
*MdGRAS80*	MDP0000321018	chr11:25863923.25875124	3,474	1157	+	130.01	−0.289	6.43	45.30	87.14
*MdGRAS81*	MDP0000768445	chr11:34920904.34922793	1,889	629	+	71.39	−0.276	5.81	48.55	80.59
*MdGRAS82*	MDP0000119093	chr12:19433725.19435980	2,256	751	+	84.11	−0.499	7.09	48.76	74.51
*MdGRAS83*	MDP0000884524	chr12:24351825.24353366	1,542	513	+	56.34	−0.119	5.36	34.40	92.22
*MdGRAS84*	MDP0000148294	chr12:24959928.24961310	1,383	460	−	52.22	−0.303	4.90	58.00	83.91
*MdGRAS85*	MDP0000235134	chr12:24972396.24973970	1,575	525	−	59.08	−0.337	4.67	53.02	75.41
*MdGRAS86*	MDP0000926466	chr12:24983309.24984916	1,608	535	−	60.29	−0.344	4.72	52.16	75.08
*MdGRAS87*	MDP0000666991	chr12:25017893.25019506	1,614	537	+	61.11	−0.347	4.69	51.34	71.55
*MdGRAS88*	MDP0000397638	chr12:26835765.26838962	2,475	824	−	88.38	−0.296	6.01	55.30	76.52
*MdGRAS89*	MDP0000266986	chr13:2853393.2854769	1,377	458	+	51.63	−0.180	5.67	53.61	94.78
*MdGRAS90*	MDP0000464809	chr13:7394453.7398805	2,856	951	+	105.52	−0.238	6.01	49.95	83.71
*MdGRAS91*	MDP0000122441	chr13:13603697.13605022	1,326	441	−	49.16	−0.013	8.38	47.79	87.82
*MdGRAS92*	MDP0000245252	chr13:13603924.13605249	1,326	441	−	49.11	−0.013	8.25	46.59	86.94
*MdGRAS93*	MDP0000522931	chr13:13606211.13607461	1,251	416	−	46.86	−0.077	5.19	37.37	90.41
*MdGRAS94*	MDP0000245253	chr13:13606548.13608350	1,803	600	−	67.20	−0.225	4.97	35.14	80.88
*MdGRAS95*	MDP0000173434	chr13:25599457.25600123	597	198	+	21.97	0.356	6.14	44.59	119.19
*MdGRAS96*	MDP0000138367	chr13:25626475.25627141	495	164	−	18.47	0.405	5.84	33.17	120.67
*MdGRAS97*	MDP0000274120	chr13:26280448.26284950	3,743	980	+	107.63	−0.315	6.78	49.41	80.11
*MdGRAS98*	MDP0000308434	chr13:30511943.30513601	1,659	552	−	61.15	−0.647	6.07	40.73	66.61
*MdGRAS99*	MDP0000431628	chr14:22609508.22611103	1,596	531	−	58.82	−0.149	5.78	56.26	87.29
*MdGRAS100*	MDP0000258655	chr14:22631424.22637535	777	258	+	29.07	−0.279	8.86	50.64	85.81
*MdGRAS101*	MDP0000259403	chr14:22631424.22637537	777	258	+	29.07	−0.279	8.86	50.64	85.81
*MdGRAS102*	MDP0000319851	chr14:28611108.28615187	2,319	772		87.26	−0.146	8.63	43.98	95.08
*MdGRAS103*	MDP0000171602	chr14:28838647.28840187	1,506	501	+	56.15	−0.388	5.37	46.69	66.99
*MdGRAS104*	MDP0000687763	chr14:28859934.28861253	1,320	440	−	49.36	−0.292	5.92	43.43	76.30
*MdGRAS105*	MDP0000840369	chr14:28859987.28861525	1,539	512	−	57.41	−0.381	5.33	47.06	68.22
*MdGRAS106*	MDP0000196258	chr14:29246984.29249224	747	248	+	27.99	−0.505	7.52	41.95	59.76
*MdGRAS107*	MDP0000662303	chr15:11412854.11414497	1,644	547	−	59.78	−0.097	5.53	42.31	92.07
*MdGRAS108*	MDP0000575908	chr15:19584108.19585916	1,809	602	+	66.40	−0.371	5.11	49.76	77.21
*MdGRAS109*	MDP0000315270	chr15:23947574.23950023	2,181	726	+	79.31	−0.283	5.26	48.40	85.76
*MdGRAS110*	MDP0000295058	chr15:25739441.25740609	1,035	344	+	38.97	−0.104	6.01	43.81	87.53
*MdGRAS111*	MDP0000187914	chr15:25748188.25749720	1,533	510	−	57.15	−0.072	6.04	45.81	89.67
*MdGRAS112*	MDP0000241137	chr15:25750590.25752014	1,425	474	+	52.94	−0.046	5.69	43.48	89.51
*MdGRAS113*	MDP0000263576	chr17:336468.348106	4,695	1564	−	175.11	−0.600	5.36	53.34	69.93
*MdGRAS114*	MDP0000441088	chr17:844678.846561	1,884	627	+	70.97	−0.272	5.51	39.89	79.60
*MdGRAS115*	MDP0000784909	chr17:4316837.4319083	2,247	747	−	81.54	−0.295	5.56	59.76	77.30
*MdGRAS116*	MDP0000827871	chr17:7181440.7182768	1,329	442	−	49.03	−0.127	5.30	46.49	91.49
*MdGRAS117*	MDP0000152670	chr17:9016383.9018038	1,656	551	−	61.95	−0.355	6.39	53.78	78.62
*MdGRAS118*	MDP0000284679	chr17:9255749.9257927	1,917	638	+	70.63	−0.136	5.32	52.66	85.00
*MdGRAS119*	MDP0000205236	chr17:9258368.9260546	1,917	638	+	70.63	−0.136	5.32	52.66	85.00
*MdGRAS120*	MDP0000289554	chr17:10806972.10811830	1,926	641	−	70.67	−0.198	9.28	48.25	90.41
*MdGRAS121*	MDP0000269044	chr17:10818117.10822975	1,926	641	−	70.67	−0.198	9.28	48.25	90.41
*MdGRAS122*	MDP0000669451	chr17:22659094.22660848	1,755	584	−	63.71	−0.281	4.91	45.60	79.73
*MdGRAS123*	MDP0000950387	chr17:22676488.22677480	993	330	−	36.58	−0.099	5.50	41.12	84.61
*MdGRAS124*	MDP0000319342	unanchored:17969995.17971509	1,515	504	−	55.32	−0.205	5.13	34.83	85.40
*MdGRAS125*	MDP0000237978	unanchored:26304735.26306654	1,920	639	−	70.24	−0.287	5.25	46.96	76.51
*MdGRAS126*	MDP0000640034	unanchored:50237359.50239266	1,908	635	+	69.73	−0.274	5.26	47.40	77.92
*MdGRAS127*	MDP0000291579	unanchored:106037882.106039530	1,593	530	+	60.97	−0.425	9.28	46.99	83.91

a Gene ID in the apple (Malus × domestica) genome (Malus domestica Genome database v1.0);

b MW: Molecular weight;

c GRAVY: Grand average of hydropathicity;

d PI: Isoelectric point;

e II: Instability index;

f *AI: Aliphatic index*.

### Statistical analysis

Data were analyzed using variance (ANOVA) at the 5% level with the SPSS 11.5 software package (SPSS, Chicago, IL, USA). Figures were made using Origin 8.0 (Microcal Software Inc., Northampton, MA, USA).

## Results

### Identification and characterization analysis of *MdGRAS* genes

**Figure 1:**
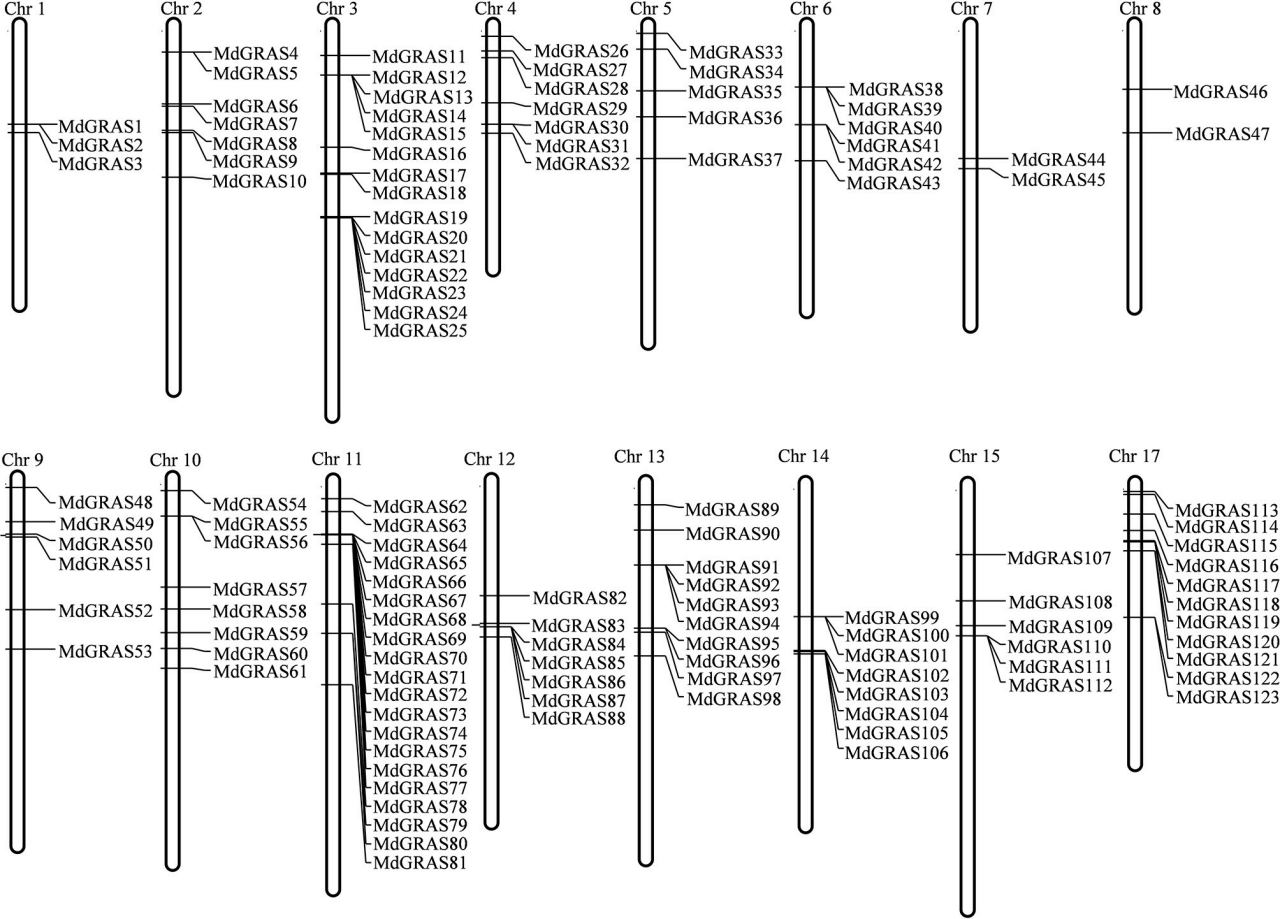
**Chromosome map of ***MdGRAS*** genes in apple**.

To identify members of the *MdGRAS* gene family, both HMM profiles and BLASTP were used with the default parameters. After manual checking, a total of 127 candidate *MdGRAS* genes were identified (Table 1). The genes were named according to their chromosomal locations. The 127 candidate *MdGRAS* genes were distributed on 16 chromosomes of the apple genome (Figure 1). Chromosome 11 contained the most *MdGRAS* genes (15.75%), followed by chromosome 3 (11.81%), while chromosome 16 contained no *MdGRAS* genes (0%) (Figure S1). Additionally, four genes (MDP0000950387, MDP0000319342, MDP0000640034, and MDP0000640034) could not be located on any of the chromosomes and were named *MdGRAS124* to *MdGRAS127*. Only two genes were distributed on each of chromosomes 7 and 8. The open reading frame (ORF) lengths of the *MdGRAS* genes ranged from 333 to 5,787 bp, encoding peptides ranging from 110 to 1,928 amino acids. We used ExPaSy to predict the characterization of MdGRAS proteins. The predicted molecular weights of the GRAS proteins ranged from 12.50 to 218.53 kDa. The grand average of hydropathicity was less than 0 for all GRAS proteins. The instability index of most of the 127 MdGRAS proteins was greater than 40, except for MdGRAS22, MdGRAS33, MdGRAS41, MdGRAS42, MdGRAS43, MdGRAS53, and MdGRAS65, indicating that these proteins were unstable.

### Phylogenetic and gene structure of the GRAS gene family

**Figure 2:**
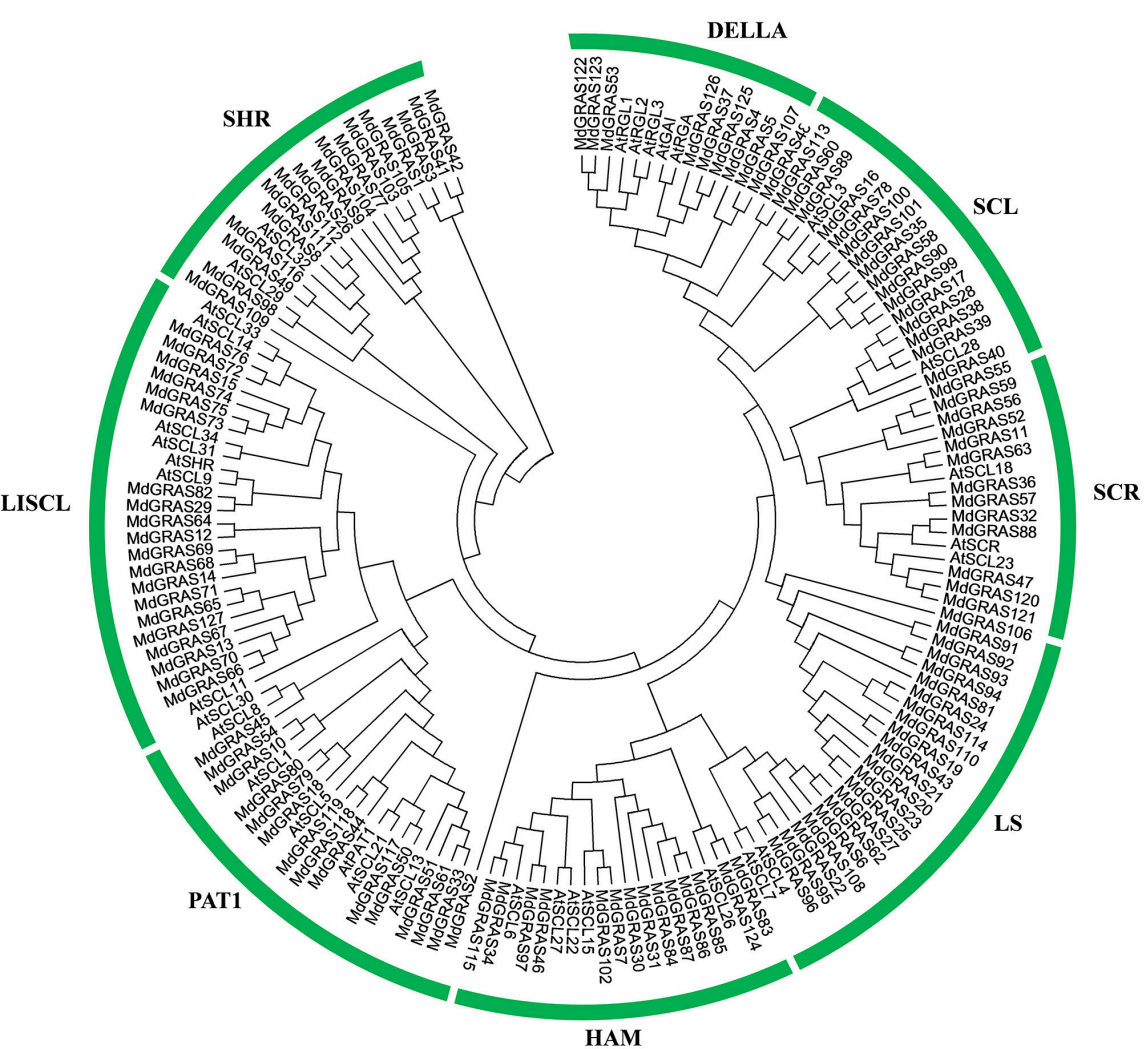
**Phylogenetic tree showing the evolutionary relationships of GRAS proteins in apple and Arabidopsis**. The phylogenetic tree was conducted using MEGA 5.10. The prefixes At and Md indicate *Arabidopsis* and apple, respectively.

Since *Arabidopsis* is the most popular model species and the functions of some GRAS genes have been well characterized. Phylogenetic analysis of *GRAS* genes was investigated in apple and *Arabidopsis*. A total of 160 GRAS sequences were collected and analyzed. They were classified into eight distinct groups according to the phylogenetic tree: DELLA, SCL3, SCR, Ls, LISCL, PAT1, SHR, and HAM (Figure 2). 14 GRAS proteins were located in the DELLA subfamily, including five AtGRAS (AtGAI, *AtRGA, AtRGL1, AtRGL2*, and *AtRGL3*) and nine MdGRAS proteins. However, MdGRAS37 did not contain the integral DELLA and TVHYNP domains. MdGRAS37 was classified into the DELLA subfamily because of its high homology with the other proteins. To examine the gene structures, we analyzed the exon-intron structures of the 127 *MdGRAS* genes according to their genome sequences (Figure S2). Thirteen genes (*MdGRAS100, MdGRAS101, MdGRAS25, MdGRAS7, MdGRAS102, MdGRAS2, MdGRAS33, MdGRAS51, MdGRAS80, MdGRAS45, MdGRAS29, MdGRAS1*, and *MdGRAS3*) had two or more introns. Other genes had few or no introns.

### Expansion patterns of the MdGRAS gene family

**Figure 3:**
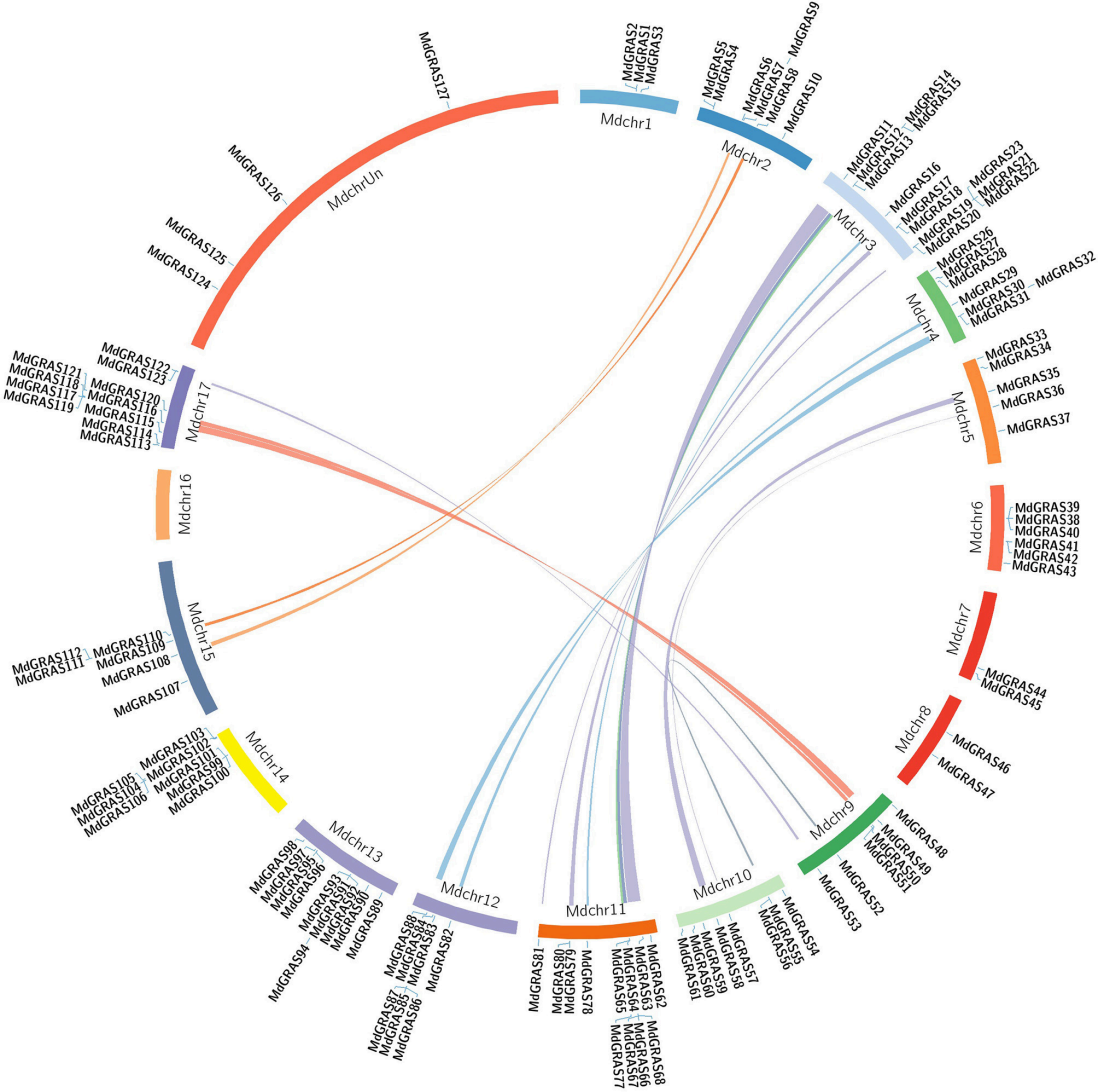
**Syntenic relationships of apple ***GRAS*** genes**. Locations of *MdGRAS* genes on the apple chromosomes.

Segmental and tandem duplications provide information on the expansion of the gene family. Here, we used the Circos program to evaluate the segmental and tandem duplications of the *MdGRAS* genes. Tandem duplications were identified from adjacent homologs on a single chromosome, while segmental duplications were identified from homologs on different chromosomes. More than 15 pairs of *MdGRAS* genes, such as *MdGRAS122*/*53, MdGRAS116*/*50*, and *MdGRAS115*/*49*, were located in duplicated genomic regions (Figure 3). All were located in different chromosomes. Chromosomes 11 and 3 had the most duplication, which could partly explain the larger numbers of *MdGRAS* genes on chromosomes 11 and 3 (Figure S1). Chromosomes 1, 6, 7, 8, 13, and 14 did not contain any duplicated genes. In summary, segmental and tandem duplications among *MdGRAS* genes have contributed to the expansion of the gene family.

### Comparative analysis of the GRAS gene families in different species

To further analyze the evolutionary relationships and expansion patterns of plant GRAS genes, we compared the published GRAS genes in different species (Table S2). The number of GRAS genes was the largest in apple and was close to that in *Populus*. However, the apple genome is twice the size of the *Populus* genome. The number of GRAS genes was also similar between Chinese cabbage (48) and *P. mume* (46), and between grape (52) and tomato (53). However, their genome sizes are also very different (Chinese cabbage: 485.0 Mb vs. *P. mume*: 280.0 Mb; grape: 487.0 Mb vs. tomato: 760.0 Mb). These results suggested that the number of plant GRAS genes was partly related to genome size.

**Figure 4:**
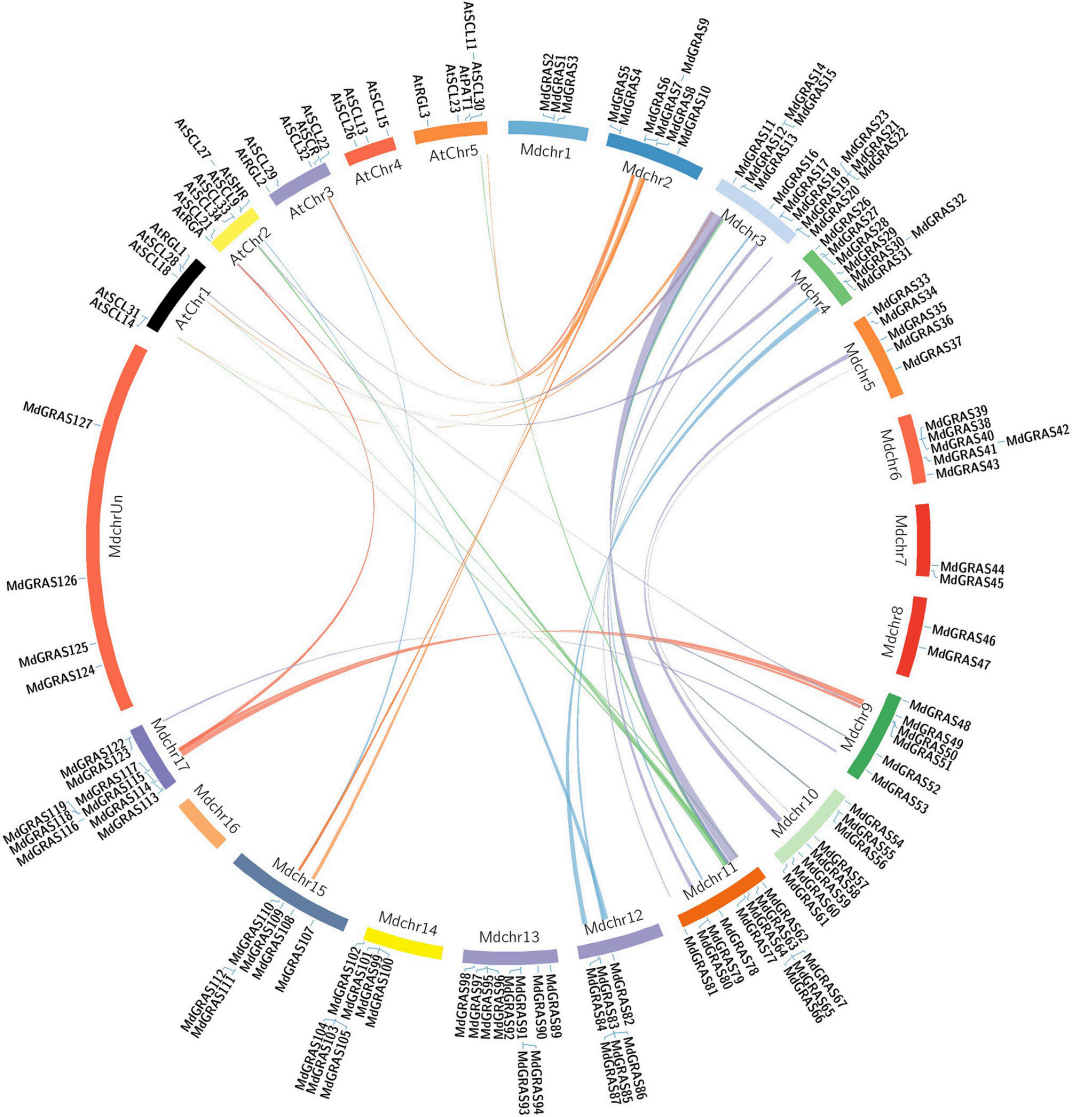
**Syntenic relationships between apple and Arabidopsis ***GRAS*** genes**. Colored curves represent apple and Arabidopsis syntenic gene regions.

As *Arabidopsis* is a model plant species, the functions of the *GRAS* genes in *Arabidopsis* have been well researched. We generated a comparative GRAS synteny map between apple and *Arabidopsis*. Many pairs of syntenic orthologous genes were matched, such as *AtSCL32*-*MdGRAS108, AtRGA*-*MdGRAS114*, and *AtPAT1*-*MdGRAS63* (Figure 4). These genes were detected on 14 chromosomes with duplicated regions, including 4 chromosomes in *Arabidopsis* and 10 chromosomes in apple.

### Tissue-specific analysis of *MdGRAS* expression using ArrayExpress data

**Figure 5:**
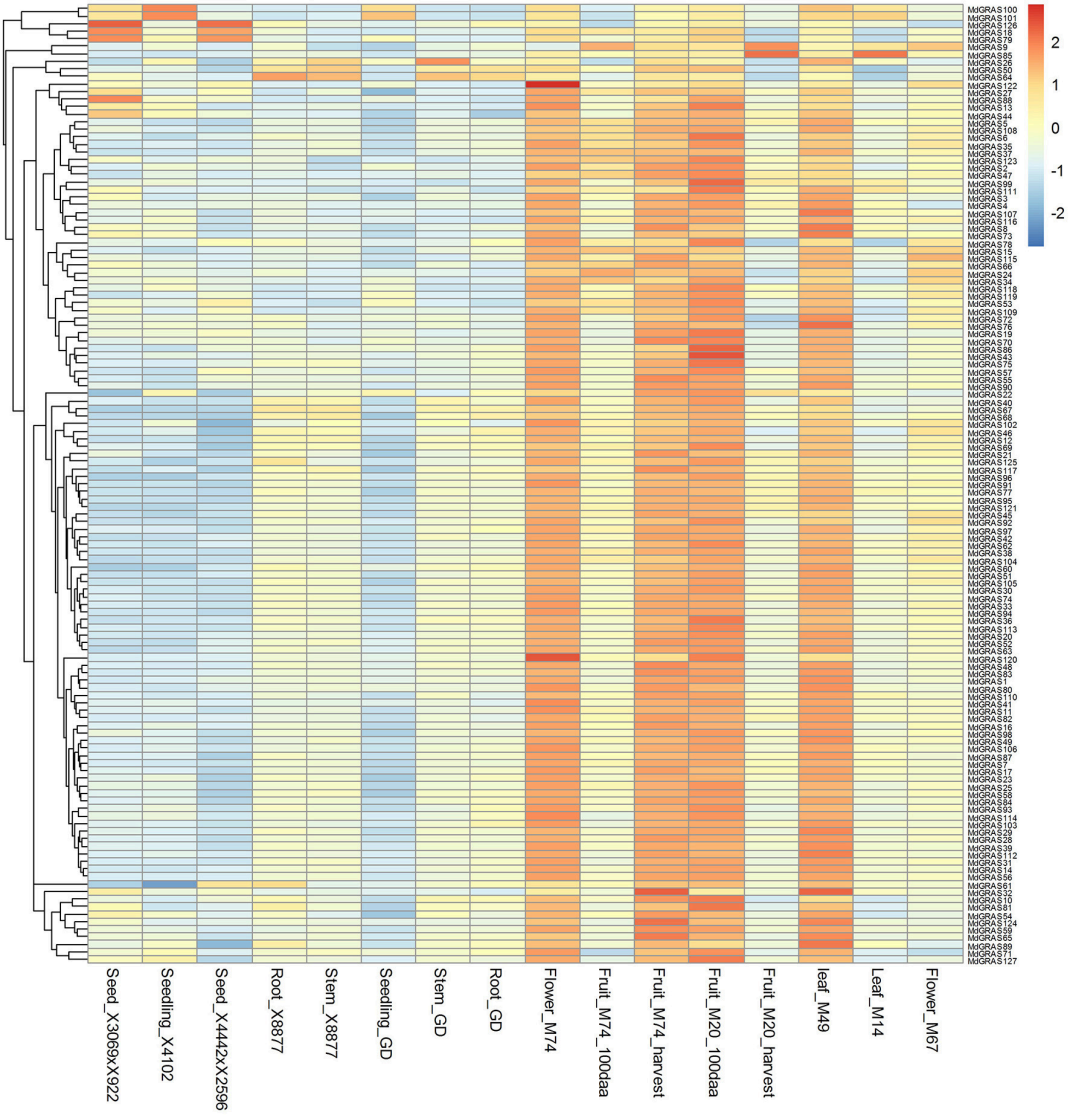
**Heat map showing ***MdGRAS*** gene transcript levels in different tissues**. Relative transcript levels are based on ArrayExpress data.

To elucidate the potential roles and functions of the *MdGRAS* genes in apple, we downloaded expression profile data for different tissues from the ArrayExpress database (E-GEOD-42873). Eight organs and samples with two biological replicates were represented in this expression array, including seedlings, roots, stems, flowers, fruit, and leaves (Figure 5). The *GRAS* genes showed different expression patterns among different apple varieties and tissues. In the “M74” apple genotype, most of the *MdGRAS* genes were expressed at higher levels in the flower than in the fruit. Moreover, the expression levels of the *MdGRAS* genes were higher in ripe fruit (at harvest) than in unripe fruit, indicating their important roles in fruit ripening. Most *MdGRAS* genes were expressed at lower levels in seedlings, except for *MdGRAS100, MdGRAS101, MdGRAS126, MdGRAS18, MdGRAS79*, and *MdGRAS27*. In “M14” leaves, *MdGRAS85* showed the highest expression. However, other *GRAS* genes were hardly detected in leaves. Moreover, most *GRAS* showed lower levels in stems except *MdGRAS26*, MdGRAS50, and *MdGRAS64*.

### QRT-PCR analysis of the candidate flowering related *MdGRAS* genes in different tissues in “Nagafu No. 2”

**Figure 6:**
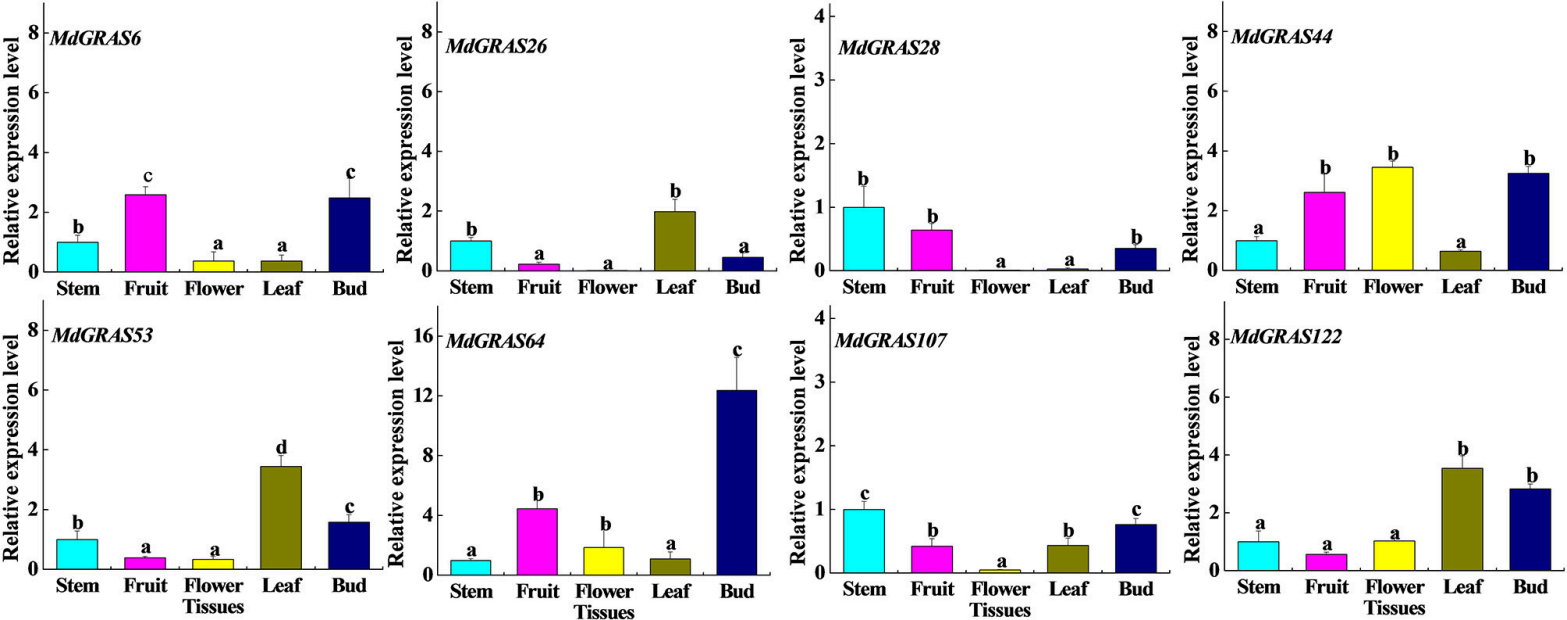
**Expression of candidate MdGRAS genes in different tissues**. Each value represents the mean Âś standard error of three replicates. Means followed by small letters (a, b, and c) are significantly different at a level of 0.05.

We selected eight higher expression genes (*MdGRAS6, MdGRAS26, MdGRAS28, MdGRAS44, MdGRAS53, MdGRAS64, MdGRAS107*, and *MdGRAS122*) from our transcriptome data for further analysis (Figure S4, Table S3). These eight genes were from different *GRAS* subfamilies, including the LS (*MdGRAS6*), SHR (*MdGRAS26*), SCL (*MdGRAS28*), PAT1 (*MdGRAS44*), DELLA (*MdGRAS53, MdGRAS107*, and *MdGRAS122*) and LISCL (*MdGRAS64*) subfamily. We employed qRT-PCR to investigate their expression patterns in different tissues (stems, leaves, flowers, fruits and buds). All candidate *MdGRAS* genes showed constitutive expression patterns in the five tissues, except that the levels of *MdGRAS26* and *MdGRAS28* were low in flowers (Figure 6). The transcription levels of *MdGRAS6, MdGRAS26, MdGRAS44, MdGRAS53, MdGRAS64, MdGRAS107*, and *MdGRAS122* were high in buds and leaves, which indicated their important roles in mediating flower induction. Additionally, *MdGRAS107* and *MdGRAS28* were all showed lower levels than other candidate genes among the five tissues.

### QRT-PCR analysis of the candidate *MdGRAS* genes in different apple varieties

**Figure 7:**
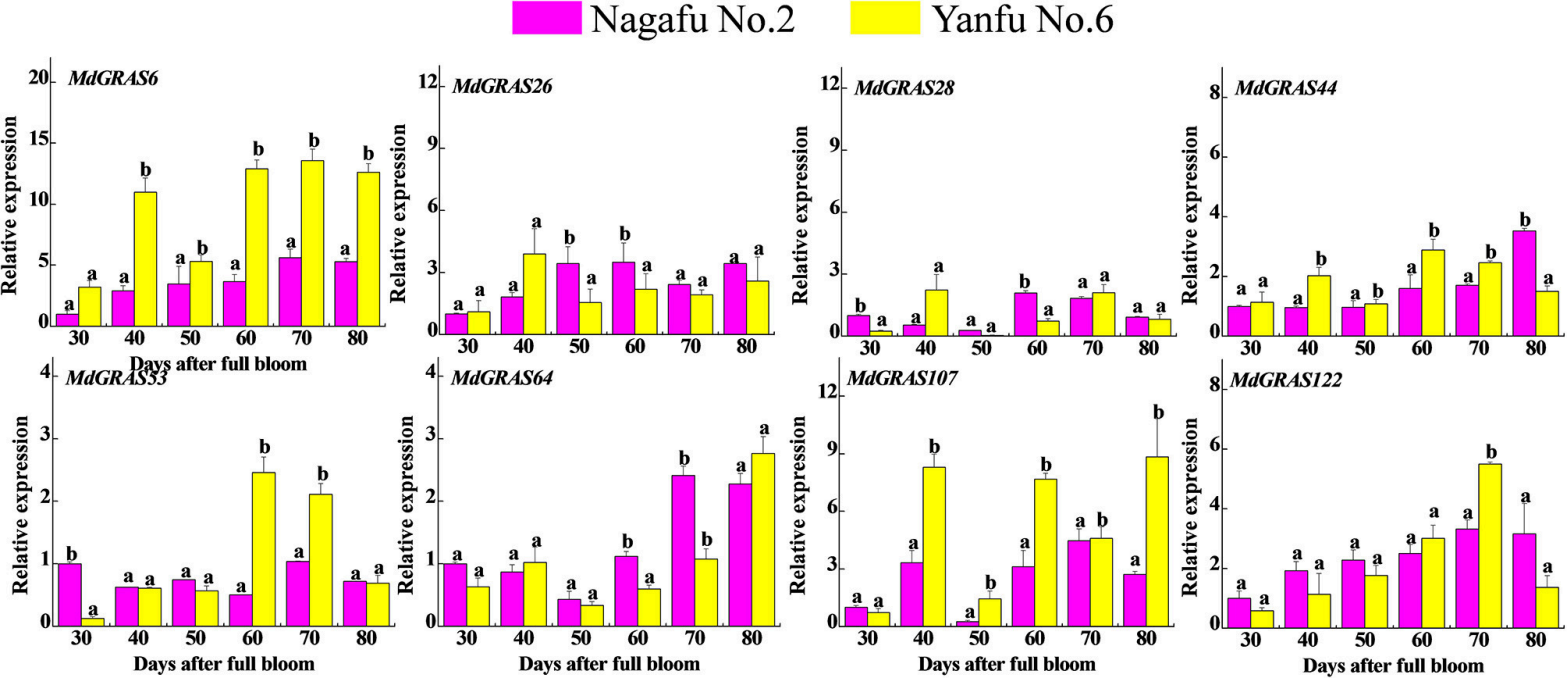
**Expression of candidate MdGRAS genes in apple buds in the easy-flowering variety “Yanfu No. 6” and different-flowering “Nagafu No. 2.”** Samples were collected at 30, 40, 50, 60, 70, and 80 days after full bloom (DAFB). Each value represents the mean ± standard error of three replicates. Means followed by small letters (a, b) are significantly different at a level of 0.05.

To examine the expression of the *MdGRAS* genes in two varieties with contrasting flowering rates, we selected the easy-flowering variety “Yanfu No. 6” and the difficult-flowering “Nagafu No. 2.” As shown in Table S4, “Yanfu No. 6” has a 1.5-fold higher flowering rate than “Nagafu No. 2.” We examined the expression patterns in these varieties using qRT-PCR (Figure 7). All candidate genes showed different expression patterns during flower induction. *MdGRAS6* expression was higher in the buds of “Yanfu No. 6” during all stages. *MdGRAS44* expression was higher in “Yanfu No. 6” except at 80 DAFB, while *MdGRAS122* expression was higher in “Nagafu No. 2” except at 70 DAFB. *MdGRAS26* expression was lower at the first stage in “Nagafu No. 2,” but was higher in all subsequent stages. The transcription level of *MdGRAS107* was higher in “Yanfu No. 6” except at 30 DAFB.

### QRT-PCR analysis of the candidate *MdGRAS* genes in response to treatments affecting flowering

**Figure 8:**
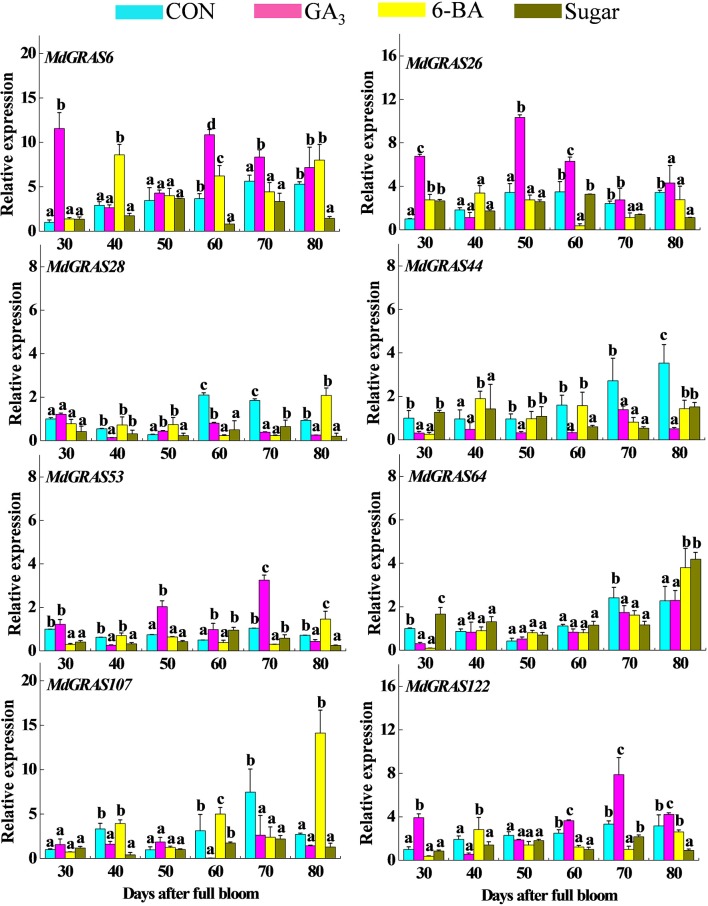
**Expression of candidate MdGRAS genes in apple buds treated with GA_**3**_, 6-BA, and sugar**. Samples were collected at 30, 40, 50, 60, 70, and 80 days after full bloom (DAFB). Each value represents the mean ± standard error of three replicates. Means followed by small letters (a, b, and c) are significantly different at a level of 0.05.

Previous studies have shown the exogenous treatments (GA, 6-BA, and sucrose) can alter flowering rates, among which GA always decreases the flowering rate, while 6-BA and sugar increase flowering rates (Xing et al., 2015; Li et al., 2016; Zhang et al., 2016). We used these kinds of treatments to further investigate the expression of the candidate *MdGRAS* genes during flowering. As shown in Table S4, exogenous GA_3_ treatment inhibited flowering rate while sugar and 6-BA promoted flowering. The genes displayed various expression patterns at different time points (Figure 8). Some of them were down-regulated, while others were up-regulated. *MdGRAS6* was up-regulated after GA_3_ treatment except at 40 DAFB and after 6-BA treatment except at 70 DAFB, but was down-regulated after sugar treatment except at 30 DAFB. *MdGRAS26* showed similar changes after GA_3_ treatment, and was up-regulated except at 40 DAFB. However, the level of *MdGRAS26* was down-regulated after 6-BA and sugar treatment except at 30 DAFB. *MdGRAS28* and *MdGRAS44* were down-regulated after GA_3_ treatment except that *MdGRAS28* was up-regulated at 30 and 50 DAFB. *MdGRAS28* was down-regulated after sugar treatment at all time points. The expression of *MdGRAS53* was promoted by exogenous GA_3_ except at 40 and 80 DAFB, but was inhibited by exogenous 6-BA and sugar at most time points. *MdGRAS64* expression was up-regulated after sugar treatment, but was not consistent in response to GA and 6-BA. *MdGRAS107* was down-regulated after GA_3_ treatment except at 30 and 50 DAFB, but was up-regulated by 6-BA except at 30 and 70 DAFB. *MdGRAS107* was down-regulated after sugar treatment except at 30 DAFB. The transcription level of *MdGRAS122* was lower in 6-BA treated buds than control except at 40 DAFB, and it was down-regulated after sugar treatment at all time points. In order to further confirm the results, we also investigated their expression patterns in GA-treated buds in 2014 and 6-BA treated buds in 2013 based on our previous research (Li et al., 2016; Zhang et al., 2016). Samples were collected in the early stage (ES), middle stage (MS), and later stage (LS) of flower induction. As shown Figures S5, S6, their expression patterns in 2014 and 2015 were similar with our results.

### Analysis related *cis*-elements in the candidate *MdGRAS* genes

To further analyze the potential roles of *MdGRAS* genes in response to various circumstances, a 1.5 kb promoter of candidate *MdGRAS* genes were used. The related *cis*-elements were identified as previous study (Fan et al., 2017). The candidate *MdGRAS* genes all shared large light-responsive boxes, followed by stress (Figure S7). Additionally, hormones-related *cis*-elements were also found in all the candidate *MdGRAS* genes, including MeJA, salicylic acid, gibberellin, auxin and ethylene. These identified motifs in the promoter of each gene indicated that *MdGRAS* may be regulated by various *cis*-elements within the promoter during growth.

## Discussion

Genome-wide identification of *GRAS* genes has been reported in *Arabidopsis, P. mume, Populus*, rice, grape, castor bean, and Chinese cabbage (Tian et al., 2004; Song et al., 2014; Huang et al., 2015; Lu et al., 2015; Grimplet et al., 2016; Xu W. et al., 2016), but little in known in apple. In this study, we identified *MdGRAS* genes in the apple genome (Velasco et al., 2010), and analyzed their phylogenetic relationships, gene characteristics, gene structures, expansion history, and syntenic relationships. We also analyzed their tissue-specific expression patterns and their expression profiles in response to flower induction. Our comprehensive study provides a basic for further investigation of *GRAS* genes in apple, which can also have potential values in genetic improvement of flower induction in apple as well as some other relative species.

### Phylogenesis of apple *GRAS* genes

We identified 127 putative *MdGRAS* genes in apple, a larger number than that reported for other plant species such as *P. mume, Populus*, rice, grape, and Chinese cabbage (Tian et al., 2004; Song et al., 2014; Huang et al., 2015; Lu et al., 2015; Grimplet et al., 2016; Xu W. et al., 2016). The larger number of *GRAS* genes in apple might be because its genome (881.3 Mb) is larger than those of *P. mume* (280.0 Mb), rice (372.0 Mb), grape (487.0 Mb), and *Brassica rapa* (283.3 Mb). The *MdGRAS* genes were located on almost all of the chromosomes except chromosome 16. The structures of most *MdGRAS* genes were similar, and most had few introns, similar to those in *P. mume* and *Populus* (Tian et al., 2004; Song et al., 2014). These findings indicated that the structures of *GRAS* genes are highly conserved among different plant species. A previous study identified six genes in the DELLA subfamily in apple, based on data in the EST database (Foster et al., 2007). In our study, nine putative genes belonging to the DELLA subfamily were identified from the apple genome (Xing et al., 2016) according to the phylogenetic tree (Figure 2). However, *MdGRAS37*, which was classified into the DELLA subfamily, lacked the conversed DELLA domain and other features of the DELLA subfamily (Figure S3), including gene length and structural domains. *MdGRAS37* was probably placed in the DELLA clade because of its high sequence similarity with *Arabidopsis* sequences of the DELLA subfamily (Lu et al., 2015). The DELLA domain of *MdGRAS37* may have gradually degenerated during the evolutionary process or its sequence may be partly similar with DELLA proteins. However, this needs to be further researched.

### Expansion and synteny of *GRAS* genes

Gene duplications can be tandem, segmental, or arise from whole genome duplication. Duplicated genes are known to play important roles in plant growth and development.

To date, various kinds of genes encoding transcription factors have been identified, and most of them have shown gene duplications, including *AP2, MADS, SPL*, and *DOF* (Zahn et al., 2005; Moreno-Risueno et al., 2007; Shigyo et al., 2007; Kumar et al., 2016). Gene duplication is important for gene family expansion (Day et al., 2003; Cannon et al., 2004; Leister, 2004). The expansion and duplication of *GRAS* genes have also been reported in *Arabidopsis, P. mume*, and Chinese cabbage (Liu and Widmer, 2014; Song et al., 2014; Lu et al., 2015). It was reported that 85% of *AtGRAS* genes have undergone segmental duplications, including two tandemly duplicated genes (*AtSCL33* and *AtSCL34*). Here, we used the Circos program to identify tandemly duplicated genes in the apple genome. Synteny analysis of the duplicated blocks in the apple genome showed that some *MdGRAS* genes were putative segmental duplicates (Figure 2). The present result was consistent with previous study in *Arabidopsis*, which *GRAS* also occurred gene duplication in apple genome. Plant *GRAS* genes were involved in various biological processes, and the expansion of the *MdGRAS* might contribute to their diversity functions in regulation plant growth and developemt. Additionally, it was reported that a genome-wide duplication (more than 50 million years ago) contributed to the expansion of the apple chromosome number, which led to a change to 17 chromosomes from nine chromosomes (Velasco et al., 2010). So the whole genome duplications promoted the expansion of *GRAS* genes in apple. This expanded by segmental duplication or whole genome duplications of *GRAS* genes in apple genome was also proved in grape and other species (Grimplet et al., 2016). Totally, the duplication and expansion of *GRAS* genes have played important roles in gene evolution, and contributed to their varied structures and functions.

To date, little functional research has been performed on rosaceous *GRAS* genes. Comparison with homologous *GRAS* genes of the model plant *Arabidopsis* can help us further analyze the *MdGRAS* genes. Comparisons of genome sequences among different species can be valuable for the reconstruction of individual gene families (Koonin, 2005). Such genome comparisons can be used to transfer annotations from a better-studied taxon (whose genome structure and functions have been elucidated) to a less-studied taxon (Lyons et al., 2008). Based on this, we inferred the functions of the less-studied *MdGRAS* genes from the better-understood *AtGRAS* genes. We used the Circos program to compare apple and *Arabidopsis*. Most *GRAS* genes were detected in syntenic genomic regions (Figure 4), allowing us to predict their functions. Currently, most *AtGRAS* genes are well understood, including *AtSCL3* (Zhang et al., 2011), *AtSHR* (Levesque et al., 2006), *AtSCL13* (Torres-Galea et al., 2006), and *AtSLR* (Aleman et al., 2008). Thus, we can analyze the potential functions of *MdGRAS* genes based on their homologs in *Arabidopsis*. However, functions predicted in this way needs to be experimentally verified.

### Expression profiles of *MdGRAS* genes in different tissues

To reveal their potential functions in apple growth and development, we employed ArrayExpress data to analyze the expression of the *MdGRAS* genes in different tissues and organs. These potential investigations of each *GRAS* can be useful to access its possible function. Their various expression patterns were consistent with their diverse gene locations, lengths and sequences. As shown in Figure 5, the expression patterns of the *GRAS* genes differed among different tissues and varieties, which is consistent with other species (Tian et al., 2004; Song et al., 2014; Huang et al., 2015; Lu et al., 2015; Grimplet et al., 2016; Xu W. et al., 2016). For example, tomato *SlGRAS18* and *SlGRAS38* transcript levels were high at the breaker and red-ripening stages, and their expression was regulated by RIN (Fujisawa et al., 2012, 2013). In another study, transcripts of 14 *SlGRAS* genes were detected during fruit ripening (Huang et al., 2015). Our analyses of *GRAS* expression profiles confirmed that most of the *MdGRAS* genes had higher transcript levels in fruit than in other apple tissues, indicating their important roles in fruit ripening. *MdGRAS9* and *MdGRAS85* expression levels were extremely high in “M20” during the harvest stage. These results suggested that *MdGRAS9* and *MdGRAS85* play important roles during fruit development; this was consistent with previous study. In *Arabidopsis*, three *AtGRAS* genes (*SCL6, SCL22*, and *SCL27*) were shown to be involved in leaf development. In the apple genotype “M14,” *MdGRAS85* transcripts were detected at high level in leaves, suggesting that this gene may share similar functions with *AtSCL6, AtSCL22*, and *AtSCL27* in leaf development, which was consistent with their close evolutionary relationships to *MdGRAS85* (Figure 2). However, *MdGRAS85* did not show high level in leaves of “M49”; the different expressions of *MdGRAS85* may be explained by their different genotypes of “M14” and “M49.” The high expression patterns of *MdGRAS126, MdGRAS18*, and *MdGRAS79* in seeds were also consistent with *PmGRAS20* and *PmGRAS36* expression, and suggested that these genes may have important roles in seed development (Lu et al., 2015). These different expression patterns of *GRAS* indicated their various functions in apple development; further functional analysis needs to be confirmed.

We also analyzed the expression of the candidate *MdGRAS* genes in different tissues (stem, leaf, flower, fruit, and bud) in “Nagafu No. 2” with qRT-PCR. The *MdGRAS* genes all showed different expression patterns among the five tissues. Leaves and buds have been used to research flower induction in *Arabidopsis* and other plants (Galvao et al., 2012; Porri et al., 2012; Xing et al., 2015; Fan et al., 2016). The candidate genes *MdGRAS6, MdGRAS26, MdGRAS44, MdGRAS53, MdGRAS64*, and *MdGRAS122* mostly showed high expression in buds and leaves, which proved their important roles in flower induction. A previous study showed that DELLA has important roles in regulating internode elongation (Bolle, 2004). We found that the expression of *MdGRAS107*, a DELLA subfamily member, was also higher expressed in the apple stem, which indicates it might also have functions in regulating internode elongation.

### Characterization of *MdGRAS* expression during flower induction

To our best knowledge, the relationship between *GRAS* and flower induction was received less attention, apart from *MdGRL2a*. Flower induction is associated with several environmental and internal factors, including temperature, hormones and photosynthesis (Galvao et al., 2012; Porri et al., 2012; Xing et al., 2015, 2016). Hormones and sugar are of the most important factors affected apple flowering, the relationship about GRAS involved in hormones or sugar remains unknown. Up to now, GA was considered to be the most associated with flower induction; 6-BA and sugar were also involved in flower induction. And they were all well applied in orchard cultivation. Additionally, our previous studies showed that exogenous treatments can alter flowering rates; GA_3_ can inhibit flowering, while 6-BA and sugar can increase flowering rates (Xing et al., 2015; Li et al., 2016; Zhang et al., 2016). These exogenous treatments mainly alter the nutrition or hormone balance and influence the expression of related genes, which inhibits (GA) or promotes (sugar and 6-BA) flowering (Xing et al., 2015; Li et al., 2016; Zhang et al., 2016). Based on this, we investigated the candidate *MdGRAS* genes in response to these flowering-related treatments (Figure 8). With their multiple functions, the candidate *MdGRAS* genes showed different expression patterns after the treatments, and were down- or up-regulated at most time points. “Yanfu No. 6,” a bud mutant of “Nagafu No. 2,” has a higher proportion of spurs, shorter internodes and a higher flowering rate than “Nagafu No. 2” (Table S4). The differential expression of the candidate *MdGRAS* genes in these two varieties with contrasting flowering rates indicated their important roles in flower induction (Figure 7).

In recent years, although the roles of *GRAS* genes in regulating plant growth and development have become better understood, little is known about the roles of *GRAS* genes in regulating flower induction in fruit trees. Several studies have shown that *GRAS* genes are important in bud growth and development. Most DELLA subfamily gene regulate flower induction by the GA pathway, and it can influence the expression of some flowering control genes (Bolle, 2004; Galvao et al., 2012; Porri et al., 2012; Xing et al., 2014, 2015; Huang et al., 2015). However, genes from other subfamilies were also associated with flower induction. *AtGRAS* genes promoted the expression of *FT* and the *della* mutant always showed earlier flowering (Galvao et al., 2012). In *Arabidopsis*, DELLA proteins were shown to inhibit flower induction, and interact with SPL to control flower induction (Shigyo et al., 2007). Previous studies showed that the expression of one-fifth of the candidate genes was increased during flower development in *P. mume*, and DELLA gene expression was also higher in tomato buds, indicating their important roles in flower induction (Huang et al., 2015; Lu et al., 2015). In our study, we selected eight important *MdGRAS* genes from transcriptome data. They belong to different subfamilies, including LS (*MdGRAS6*), SHR (*MdGRAS26*), SCL (*MdGRAS28*), PAT1 (*MdGRAS44*), DELLA (*MdGRAS53, MdGRAS107*, and *MdGRAS122*) and LISCL (*MdGRAS64*). Our result suggested that these candidate *MdGRAS* genes from different subfamilies were important and they all have potential involvement in flower induction. Additionally, functions of their homologous genes have been described and verified in other species, which provided more evidence for their important response to flower induction (Fu et al., 2001; Hynes et al., 2003; Petty et al., 2003; Wang et al., 2010; Galvao et al., 2012). They can regulate flower induction directly and indirectly. In *Arabidopsis, AtLAS* and *AtSHR* were involved in shoot apical meristem. *AtSCL6, AtSCL26*, and *AtSCL27* were highly expressed in flowers (Bolle, 2004); the *Arabidopsis rga* and *gai* mutant showed earlier flowering (Galvao et al., 2012). Additionally, *GRAS* genes in other species were all indicated their potential involvement in flowering induction, such as tomato (*SlGRAS11* and *SlGRAS18*), castor beans (*27568.m000253, 28650.m000187, 28650.m000190, and 28650.m000191*), and grape (*VviSHR1* and *VviPAT6*) (Huang et al., 2015; Grimplet et al., 2016; Xu W. et al., 2016). Our candidate *MdGRAS* genes were homologous with these well-known *GRAS* genes in other plants, which is in agreement with their important functions in flower induction. However, further functional research needs to be confirmed about these *MdGRAS* genes. Their different expression patterns during flower induction indicated they were involved in GA-mediated, 6-BA mediated or sugar-mediated flowering in apple trees. Previous transgenic *Arabidopsis* of *MdRGL2a* (one of the DELLA members in apple) showed delayed flowering phenotype, which proved their functions in regulating flowering. Additionally, the predication of *cis*-elements within their promoters indicated their potential roles in response to hormone mediated signal during apple growth and development (Figure S7). However, with the large number of *GRAS* gene in *Arabidopsis* and apple genome, we cannot capture more genes aiming at flower induction except the candidate eight *MdGRAS*. The other left *MdGRAS* genes need to be further confirmed. And more *GRAS* genes need to be functionally researched.

All in all, extensive cross-talk about GRAS genes has been identified in herbaceous and woody plants; extensive research effort has also been devoted to characterization GRAS genes in plant growth and development. However, the GRAS genes of woody economically important tree species have received less attention. In this study, we identified 127 putative *GRAS* genes from the apple genome, which were located on 16 chromosomes. We classified these genes and performed systematic phylogenetic, structural and synteny analyses. We also analyzed their expression in different tissues (stems, leaves, flowers, fruits and buds). The spatiotemporal expression patterns indicated they potentially have multiple functions in regulating plant growth and development. Additionally, some candidate *MdGRAS* genes were investigated under treatments with several flowering-related compounds, which indicated the candidate *MdGRAS* genes are involved in the complex flower induction process. These results may provide useful strategies for further improvement of flower induction in apple.

## Author contributions

MH, SF, and DZ conceived and designed the experiment. SF, CG, and MZ performed the experiment. SF, HW, YL, and YS analyzed the data. MH and SF wrote the manuscript.

### Conflict of interest statement

The authors declare that the research was conducted in the absence of any commercial or financial relationships that could be construed as a potential conflict of interest.

## Supplementary material

Figure S1 **Distribution of ***MdGRAS*** genes on the apple chromosomes**.

Figure S2 *****MdGRAS*** gene structures**. *MdGRAS* gene exon–intron composition. Red boxes and black lines represent exons and introns, respectively.

Figure S3 **Alignment of DELLA subfamily members in apple and ***Arabidopsis*****.

Figure S4 **Heat map showing the transcript levels of ***MdGRAS*** genes during flower induction**. Transcript levels of *GRAS* genes in the buds of “Nagafu No. 2” and “Yanfu No. 6” were investigated during flower induction. Samples were collected three times at the early stage (ES), middle stage (MS) and later stages (LS) of flower induction.

Figure S5 **Expression of candidate ***MdGRAS*** genes in GA-treated buds**. Samples were collected three times at ES, MS and LS in 2014, which was consistent with 40, 50, and 70 DAFB in 2016. Each value represents the mean ± standard error of three replicates.

Figure S6 **Expression of candidate ***MdGRAS*** genes in 6BA-treated buds**. Samples were collected three times at ES, MS and LS in 2013, which was consistent with 30, 50, and 80 DAFB in 2016. Each value represents the mean ± standard error of three replicates.

Figure S7 **Predicted ***cis***-elements in the promoter regions of the candidate ***MdGRAS*** genes**. The upstream 1.5 kb promoter sequences were analyzed.

Table S1 **Primer sequences of ***MdGRAS*** and reference genes in quantitative reverse transcription PCR**.

Table S2 **Comparison of ***MdGRAS*** genes in different species**.

Table S3 **Expression levels of ***MdGRAS*** genes in Nagafu No. 2 and Yanfu No. 6**. Data were collected from the transcriptome.

Table S4 **Investigation of flowering rate in 2016**.

## Abbreviations

DAFB: days after full blossom
ES: early stage
MS: middle stage
LS: later stage
GA_3_: gibberellic acid
6-BA: 6-benzylaminopurine.
