# Supplementary figures and images for: Identification, Classification, and Expression Analysis of GRAS Gene Family in Malus domestica
Source: Front Physiol. 2017 Apr 28;8:253. doi: 10.3389/fphys.2017.00253 (PMC5408086; doi:10.3389/fphys.2017.00253)

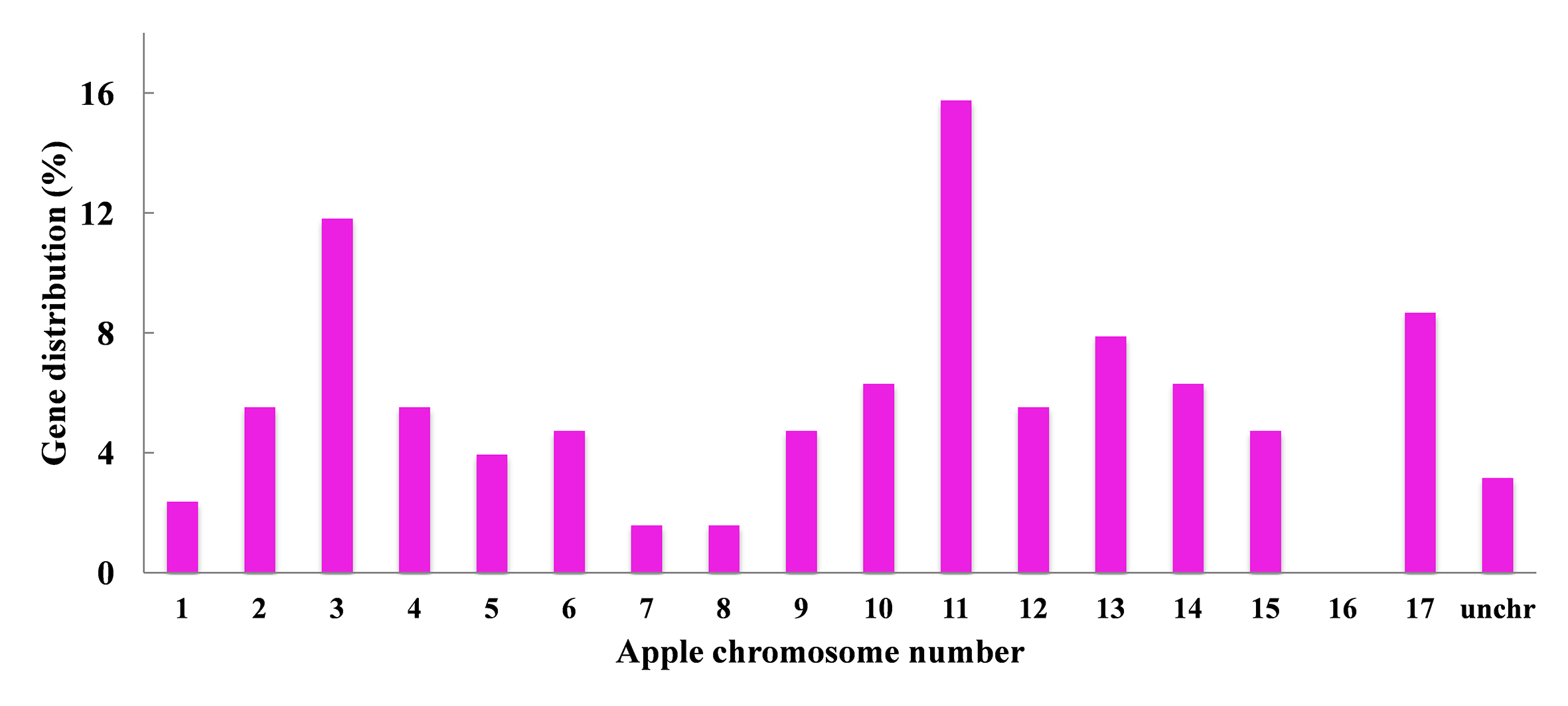

Supplement: Figure S1 — Distribution of MdGRAS genes on the apple chromosomes. [file Image1.TIF]

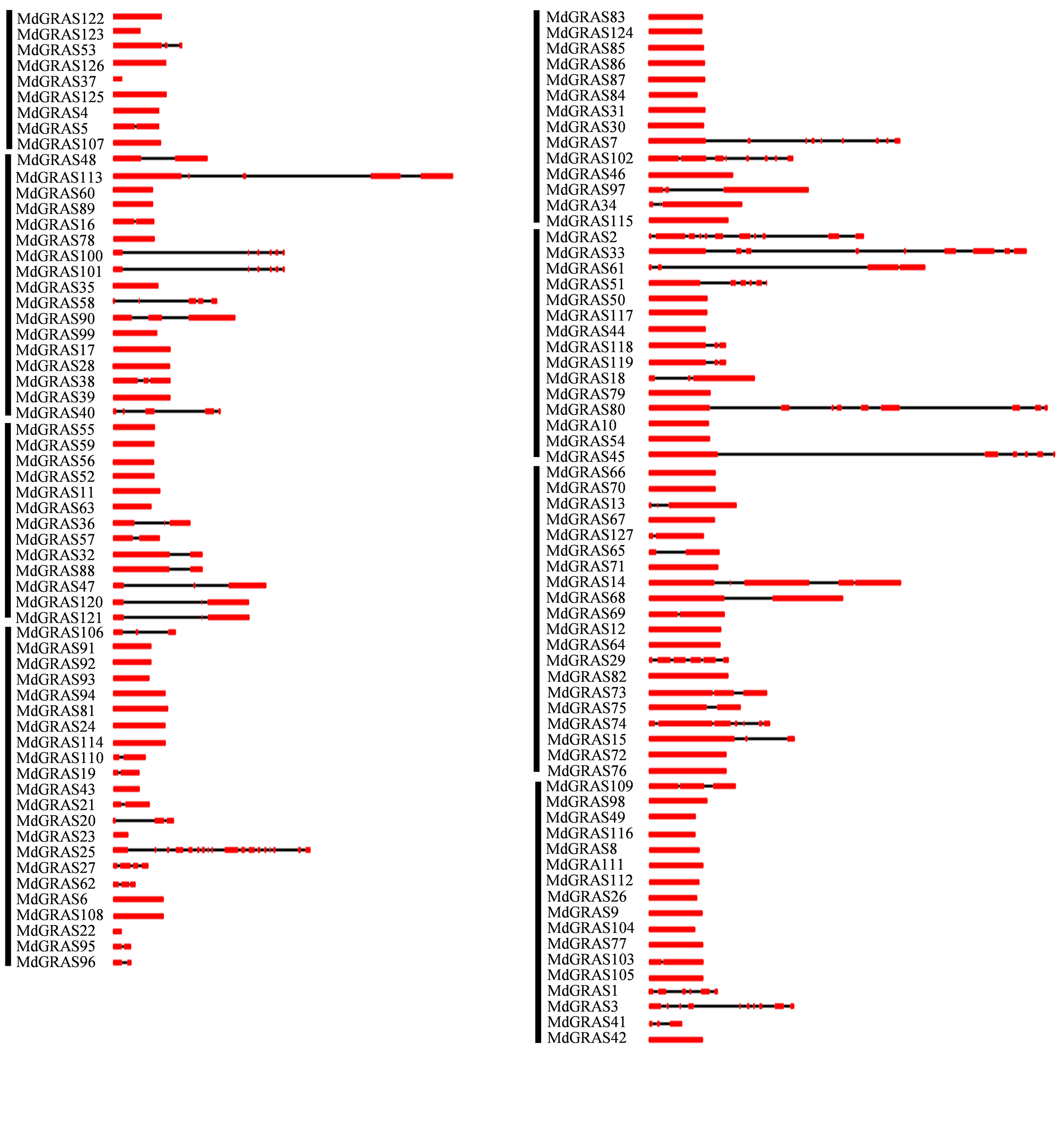

Supplement: Figure S2 — MdGRAS gene structures. MdGRAS gene exon–intron composition. Red boxes and black lines represent exons and introns, respectively. [file Image2.TIF]

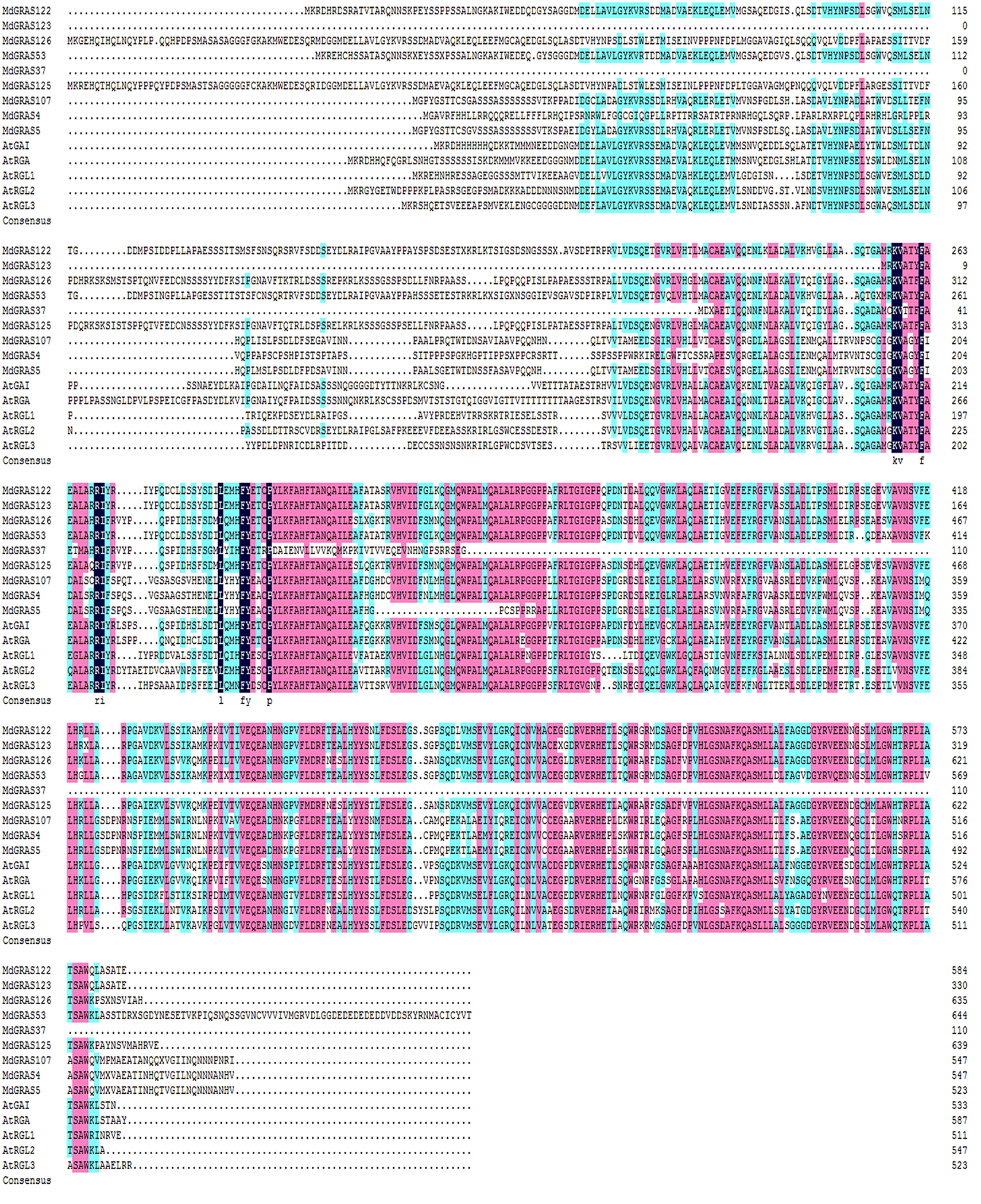

Supplement: Figure S3 — Alignment of DELLA subfamily members in apple and Arabidopsis. [file Image3.TIF]

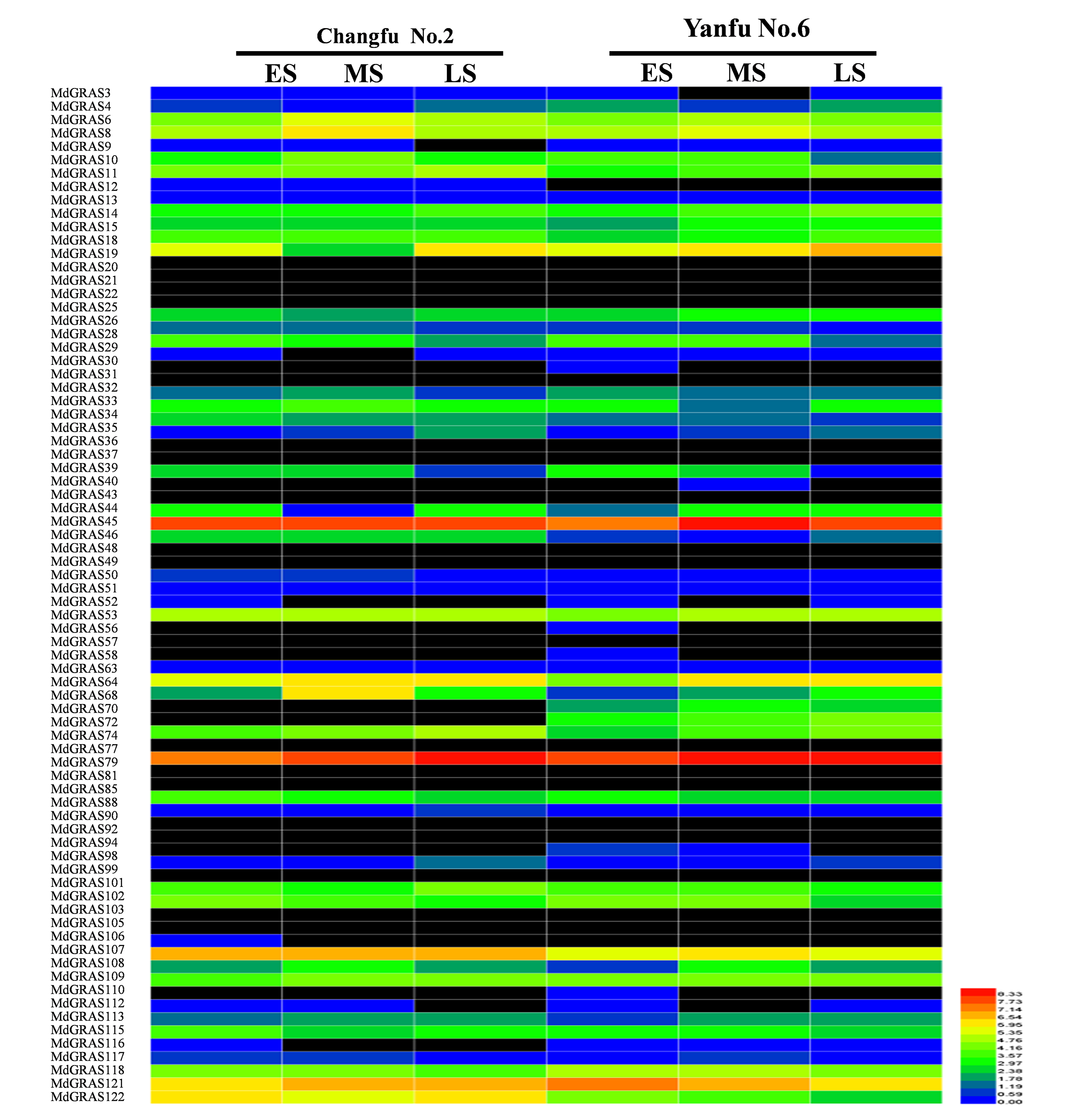

Supplement: Figure S4 — Heat map showing the transcript levels of MdGRAS genes during flower induction. Transcript levels of GRAS genes in the buds of “Nagafu No. 2” and “Yanfu No. 6” were investigated during flower induction. Samples were collected three times at the early stage (ES), middle stage (MS) and later stages (LS) of flower induction. [file Image4.TIF]

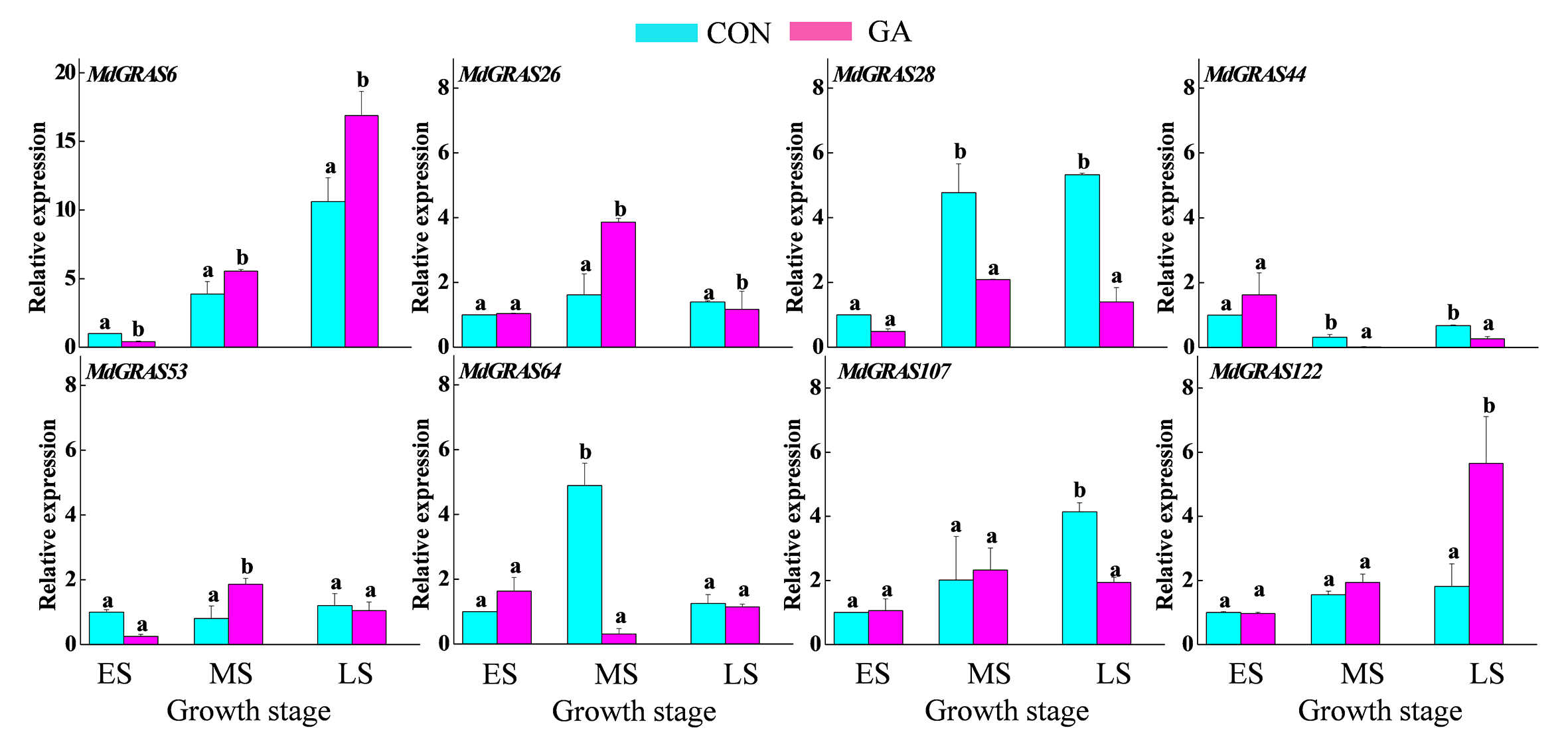

Supplement: Figure S5 — Expression of candidate MdGRAS genes in GA-treated buds. Samples were collected three times at ES, MS and LS in 2014, which was consistent with 40, 50, and 70 DAFB in 2016. Each value represents the mean ± standard error of three replicates. [file Image5.TIF]

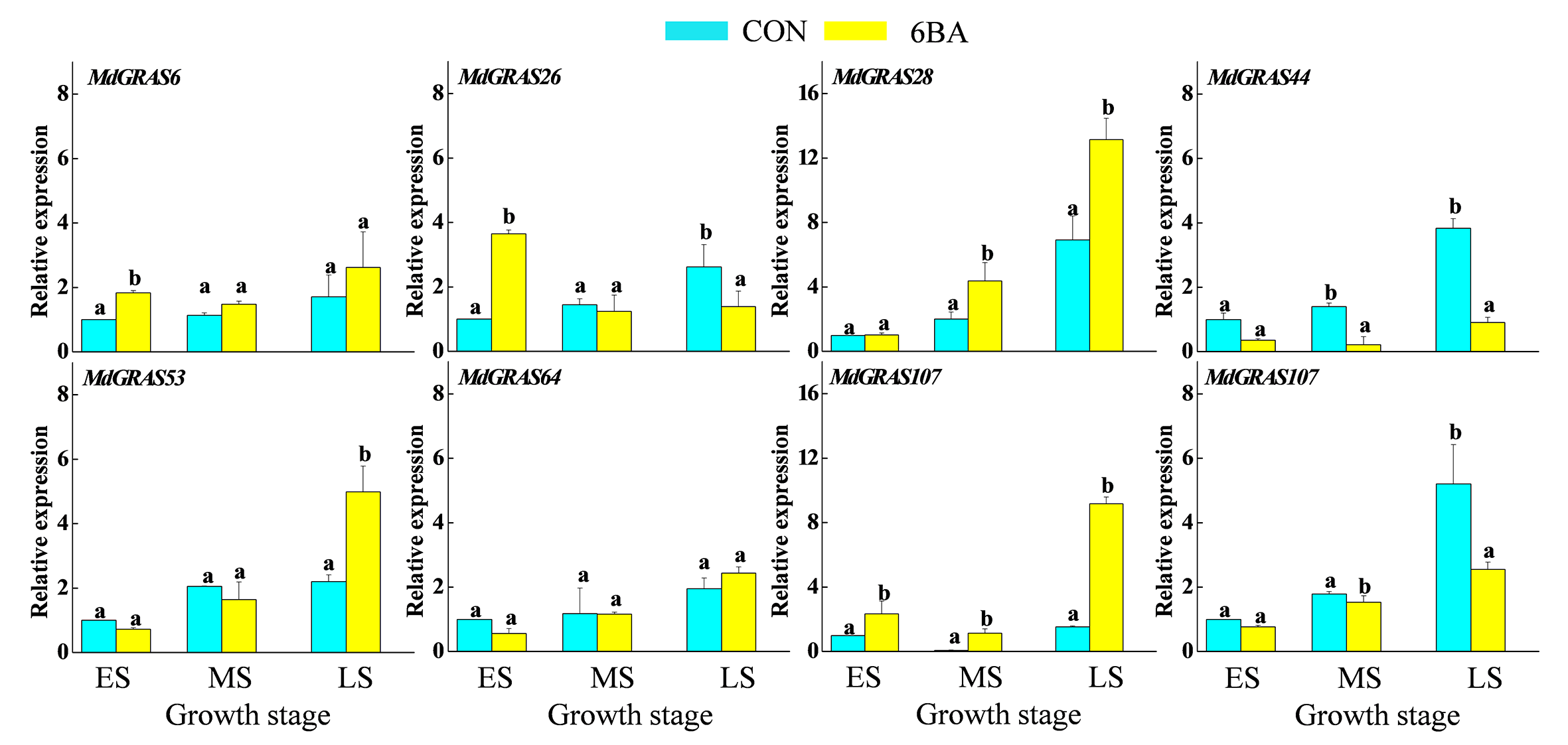

Supplement: Figure S6 — Expression of candidate MdGRAS genes in 6BA-treated buds. Samples were collected three times at ES, MS and LS in 2013, which was consistent with 30, 50, and 80 DAFB in 2016. Each value represents the mean ± standard error of three replicates. [file Image6.TIF]

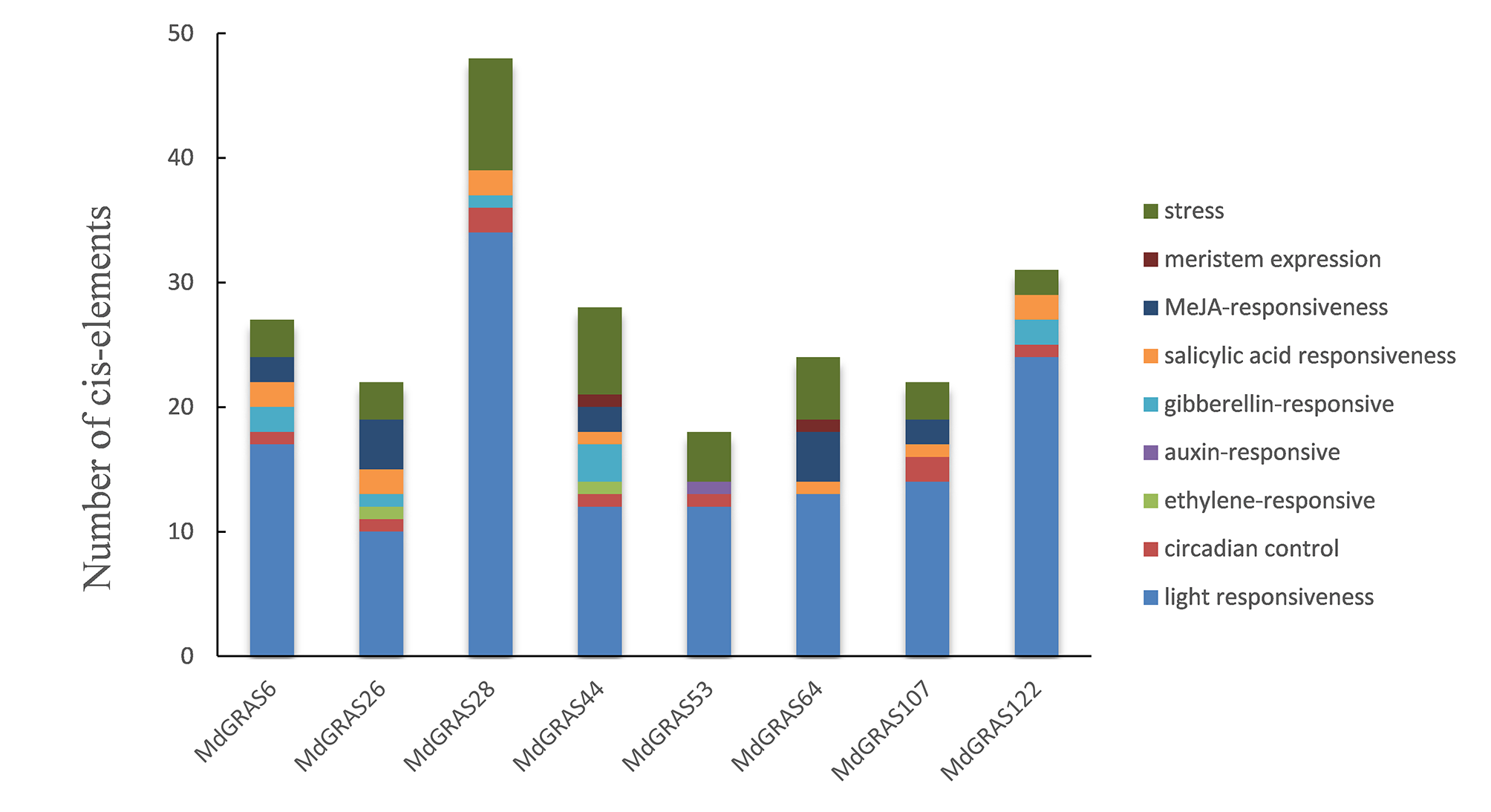

Supplement: Figure S7 — Predicted cis-elements in the promoter regions of the candidate MdGRAS genes. The upstream 1.5 kb promoter sequences were analyzed. [file Image7.TIF]
