# Supplementary material for: Identification, Classification, and Expression Analysis of GRAS Gene Family in Malus domestica
Source: Front Physiol. 2017 Apr 28;8:253. doi: 10.3389/fphys.2017.00253 (PMC5408086; doi:10.3389/fphys.2017.00253)
Supplement: Table S1 — Primer sequences of MdGRAS and reference genes in quantitative reverse transcription PCR. [file Table1.DOCX]

**Table S1. Primer sequence of MdGRAS and reference genes in quantitative real-time PCR.**

| Gene | Forward primer (5’-3’) | Reverse primer (5’-3’) | Size^a^ | Tm^b^ |
| --- | --- | --- | --- | --- |
| *MdGRAS6* | AGCAGGAGCAGCAGCAGCAA | TGTCGCAAGCAGAAGGCAGGTT | 351 | 60 |
| *MdGRAS26* | CGGTTGGATTCAGCGACGATGTTG | CCTCCTCCTTCTCCTCCTCCTTGT | 112 | 60 |
| *MdGRAS28* | CATCTCCCTCCAACGACGACTCAG | ATCCTCCTCCTCCTCTGCCACAA | 354 | 60 |
| *MdGRAS44* | GTGGTTACCCTGGTCGCAGTTCT | GGTTGACAGGAGAGCCACAAGACT | 111 | 60 |
| *MdGRAS53* | ACCGAGTTGAGCGACACGAGAC | CCCGAGCATGAGAGACCCATTGTT | 176 | 60 |
| *MdGRAS64* | ACCCAGGCAGTAATCACAGCAGTT | AGGGTTTGGAATTTGGGCAGGAGA | 116 | 60 |
| *MdGRAS107* | TAGGCTGGTCCACTTGCTTGTCA | ACGGTCTGCGGCGAGAAGATT | 186 | 60 |
| *MdGRAS122* | GGAATACTCATCACCGCCGTCTTC | ATGTCGTCGGACCGAACCTTGTA | 138 | 60 |
| *EF-1α* | ATTCAAGTATGCCTGGGTGC | CAGTCAGCCTGTGATGTTCC | 174 | 60 |

^a^ Size means the product length (bp).

b Tm means melting temperature (°C).

**Table S2 Summary of the GRAS genes of different species**

| **Species** | **Number of**  **GRAS genes** | **Genome Size**  **(Mb)** | **Number/Mb** | **Reference** |
| --- | --- | --- | --- | --- |
| Arabidopsis thaliana | 32/34 | 135.0 | 0.237 | 24,34 |
| Rice | 57/60 | 372.0 | 0.153 | 24,34 |
| Chinese cabbage | 48 | 485.0 | 0.099 | 23 |
| Populus | 106 | 422.9 | 0.251 | 34 |
| Tomato | 53 | 760.0 | 0.070 | 21 |
| *Prunus mume* | 46 | 280.0 | 0.161 | 22 |
| Grape | 52 | 487.0 | 0.107 | 20 |
| Apple | 127 | 881.3 | 0.144 |  |

**TABLE S4| Investigation of flowering rate in 2016.**

| Treatment | Control^a^ | GA3 | 6-BA | Sugar | Yanfu No.6 |
| --- | --- | --- | --- | --- | --- |
| Flowering rate (%) | 45.95±2.63b | 21.12±3.12a | 61.56±3.45c | 59.35±3.2c | 73.43±3.30d |

^a^Changfu No.2 was used as control tress between treatments and different varieties.

Each value represents the mean ± standard error of three replicates; means followed by small letters (a, b and c) are significantly different at level of 0.05.
